# Social behavioral changes in MPTP-treated monkey model of Parkinson's disease

**DOI:** 10.3389/fnbeh.2015.00042

**Published:** 2015-02-26

**Authors:** Elodie Durand, Odile Petit, Léon Tremblay, Cédric Zimmer, Véronique Sgambato-Faure, Carine Chassain, Marlène Laurent, Bruno Pereira, Céline Silberberg, Franck Durif

**Affiliations:** ^1^Université d'Auvergne Clermont 1, UFR Médecine, EA 7280 (NPsy-Sydo)Clermont-Ferrand, France; ^2^Département Ecologie, Physiologie et Ethologie, Institut Pluridisciplinaire Hubert Curien, UMR 7178, CNRS-UDSStrasbourg, France; ^3^Centre de Neurosciences Cognitives, UMR 5229 CNRS-Université Lyon 1Bron, France; ^4^CHU Clermont Ferrand, Gabriel MontpiedClermont-Ferrand, France

**Keywords:** Parkinson's disease, social behavior, non-human primate model, MPTP, PET imaging

## Abstract

Parkinsonian patients experience not only the physical discomfort of motor disorders but also the considerable psychological distress caused by cognitive deficits and behavioral disorders. These two factors can result in a disruption of social relationships during the symptomatic and even the presymptomatic motor states of the disease. However, it remains difficult, if not impossible, to evaluate social relationships in presymptomatic patients. The present study focused on the evaluation of social relationships within a group of female long-tailed macaques during presymptomatic and symptomatic motor states induced by Chronic Low-Dose (CLD) and then Chronic High-Dose (CHD) systemic administration of 1-methyl-4-phenyl-l,2,3,6-tetrahydropyridine (MPTP). Dopaminergic denervation within basal ganglia and cortical areas was evaluated using Positron Emission Tomography (PET) scans with ^18^F-DOPA (6-[18F]-fluoro-L-3,4-dihydroxyphenylalanine) radiotracer. Interestingly, social behavioral changes could be identified in the presymptomatic motor state before any motor and/or cognitive impairment occurred. Stronger effects were observed in subordinate animals compared to dominant animals. From baseline state to CLD-presymptomatic motor state, the frequency of emitted affiliative and aggressive behaviors increased. From CLD-presymptomatic to CHD-presymptomatic motor states, the frequency of the three categories of social behaviors (aggressive, submissive and affiliative) decreased. At this time, quantitative data analysis in PET scans highlighted a dopaminergic denervation in the insula and the posterior caudate nucleus. Finally, the frequency of the three categories of social behaviors decreased during the stable-symptomatic motor state compared to baseline and presymptomatic motor states; this was also associated with motor and cognitive disorders and a dopaminergic denervation in all the evaluated cortical and subcortical structures.

## Introduction

Idiopathic Parkinson's disease (PD) is characterized by a loss of dopaminergic neurons in the substantia nigra pars compacta (SNc), resulting in decreased levels about 60% of dopamine release in the striatum and hence causing motor symptoms (bradykinesia, tremor and rigidity) (Kish et al., 1988). However, it is clear that PD is not only a “motor” but also a “cognitive” and “neuropsychiatric” disease (Thobois et al., 2010; Gallagher and Schrag, 2012). Psychiatric disorders include apathetic state (Pedersen et al., 2009; Thobois et al., 2010), anxiety (Gallagher and Schrag, 2012) and depression (Martínez-Martín and Damián, 2010) as well as hypomania, psychosis and impulse control disorders observed in patients receiving dopaminergic treatment (Weintraub et al., 2006; Ulla et al., 2012). The pathophysiology of such disorders has not yet been completely understood but includes lesions of the dopaminergic, serotoninergic, and noradrenergic systems involved in Parkinson's disease (Hirsch et al., 2003; Kish et al., 2008). However, a change in the mesocorticolimbic dopaminergic system could play a role in the behavioral disorders of PD (Remy et al., 2005). Indeed, in addition to the lesion of the dopaminergic nigrostriatal system, other dopaminergic systems are also damaged in PD, namely those originating in the ventral tegmental area (VTA) that project to the limbic system, which is involved in the reward circuit (mesolimbic system), and also to the prefrontal cortex, which is involved in personality traits (mesocortical system) (Tzschentke, 2001; Haber and Knutson, 2010).

The aforementioned non-motor symptoms could have an early impact on the social life of parkinsonian patients, leading some of them to become socially isolated both from themselves and from society. The symptoms impact the quality of life of parkinsonian patients, namely affecting social interactions, communication and/or emotion recognition (Schrag et al., 2000; Yoshimura et al., 2005; Pell et al., 2006).

It is therefore legitimate to ask whether a lesion of the dopaminergic system could affect social interactions. What types of interactions could be affected by a dopaminergic lesion? Finally, which brain regions (basal ganglia and/or cortical areas) may be involved in social interactions changes? Answering these questions in patients is problematic, as it would be difficult if not impossible to accurately evaluate their social interactions on a daily basis and understand the real social impact of this disease. In this context, the use of a non-human primate model therefore appears useful to study the social impact of dopamine neuron loss in Parkinson's disease. In the wild, *Macaca fascicularis* females remain within their native group throughout their lives and therefore form clans of related individuals. Their ranks are more stable than those of males and are transmitted from mother to daughter forming a matrilineage that rarely changes over generations (Van Noordwijk and Van Schaik, 1987, 1999; Gumert, 2010). Any change in social behavior in a group of *Macaca fascicularis* might therefore impact the balance of inter-individual social relationships.

Several previous studies in non-human primates have shown the important role of several monoamines, such as dopamine, in hierarchical status and the expression of social behaviors in macaques (Redmond et al., 1971; Kaplan et al., 2002; Morgan et al., 2002; Riddick et al., 2009; Nader et al., 2012). Furthermore, neurobiological studies in non-human and human primates have also shown the role of cortico-limbic regions (namely the amygdala, the orbitofrontal cortex, the anterior cingular cortex and the insula) in the social behavioral network and in social cognition generally, which is heavily dependent upon the expression and recognition of emotions (Amaral, 2002; Machado and Bachevalier, 2006; Rushworth et al., 2007; Machado et al., 2009).

Currently, the primate model produced with the neurotoxin 1-methyl-4-phenyl-l,2,3,6-tetrahydropyridine (MPTP) is considered as the gold standard animal model of PD because of its close resemblance to PD. Indeed, this model, particularly with systemic MPTP administration in macaques, characteristically replicates marked cellular loss in the SNc, the cardinal motor symptoms of PD (including abnormalities in axial movements and postures), and the full extent of motor complications associated with chronic dopaminergic treatment (Jenner, 2003). Furthermore, the primate MPTP model can reproduce non-motor symptoms of PD including cognitive, sleep and gastrointestinal dysfunction (Barraud et al., 2009; Chaumette et al., 2009; Schneider et al., 2010). The chronic low-dose (CLD)-MPTP model of parkinsonism in non-human primates was specifically developed to study the presymptomatic motor state in early PD (Schneider and Kovelowski, 1990). This model induces a dopaminergic denervation in the SN/VTA (Schneider, 1990), dopaminergic frontostriatal cognitive deficits and deficits in spatial delayed response, set shifting, planning and impulsivity (Schneider and Kovelowski, 1990; Schneider and Roeltgen, 1993; Schneider and Pope-Coleman, 1995; Decamp and Schneider, 2004; Schneider, 2006; Vezoli et al., 2011) and recently sleep-wake disorders (Videnovic et al., 2014). Moreover, this CLD-MPTP model has been shown to induce a striatal denervation pattern more similar to the one observed in PD patients (Gibb and Lees, 1991; Perez-Otano et al., 1994) than the one induced by chronic high-dose or acute MPTP intoxication. Most previous studies using chronic high-dose administration of MPTP (CHD-MPTP), mainly focused on motor and cognitive disorders related to neurochemical, imaging or pharmacological findings (Chassain et al., 2001; Madras et al., 2006; Blesa et al., 2012; Neumane et al., 2012; Kortekaas et al., 2013). Interestingly, a longitudinal behavioral study in MPTP-hemiparkinsonian vervet monkeys also showed a link between changes in social behaviors (aggressive and affiliative) and striatal dopamine levels measured by ^18^F-DOPA (6-[18F]-fluoro-L-3,4-dihydroxyphenylalanine) PET (Positron Emission Tomography) scanning (Melega et al., 1996).

Therefore, the aim of the present study was to identify the impact of chronic exposure to low and then to high doses of MPTP on the social behavior of six female long-tailed macaques living in a social group, and to assess the time course evolution of cognitive and motor disabilities and social behavior disorder(s). PET (Positron Emission Tomography) scanning was used to assess dopaminergic denervation in basal ganglia and cortical areas using ^18^F-DOPA (6-[18F]-fluoro-L-3,4-dihydroxyphenylalanine).

## Materials and methods

### Ethics statement

All procedures were carried out according to National Institute of Health and the European Directive 2010/63/EU guidelines and the Department of Veterinary Services (DDSV Clermont Ferrand, France). These experiments were also carried out according to guidelines published in the Guide for the Care and Use of Laboratory Animals of the National Institutes of Health. Specific authorization covering this study was delivered by the regional animal ethical committee (Comité d'Ethique en Expérimentation Animale Auvergne, C2EA-02) under Permit Number: CE19-08.

### Animals

Experiments were conducted on six female long-tailed macaques (*Macaca fascicularis*) (5.2–6.6 years old, weighing 3.6–5.6 kg at the beginning of the study): monkeys A, B, C, D, E, and F. This number of animals was sufficient for statistical analysis of social behaviors. They were housed together throughout the study, had free access to water and received food twice a day. No animals were sacrificed during the study. Standard conditions of humidity (55 ± 10%), temperature (24 ± 2°C) and light (12-h light/dark cycles) were respected. The housing consisted of two rooms connected by a trap door that was left open at all times, each room consisted of three 1.8 m^3^ cages (1 × 1 × 1.8 m). Each animal therefore had access to a minimum of 1.8 m^3^, as required by National Institute of Health and the European Directive 2010/63/EU guidelines.

### Contraceptive implant

Several studies have already demonstrated an effect of menstrual cycle on social behavior (Adams et al., 1985; Michael and Zumpe, 1993; Czoty et al., 2009). In order to remove any such influence, all animals (*n* = 6) were fitted with the subdermal contraceptive implant (half an implant/animal), Implanon® (Schering-Plough, USA) primarily used in humans (Isley, 2010) prior to all testing sessions and the evaluation of baseline conditions. This implant overcomes the effects of menstrual cycles on observed behaviors and monoamines. Each Implanon® rod consists of an ethylene vinylacetate copolymer core, containing 68 mg of the synthetic progestin etonogestrel and produces an effect for 3 years.

### Toxin administration

**Figure 1:**
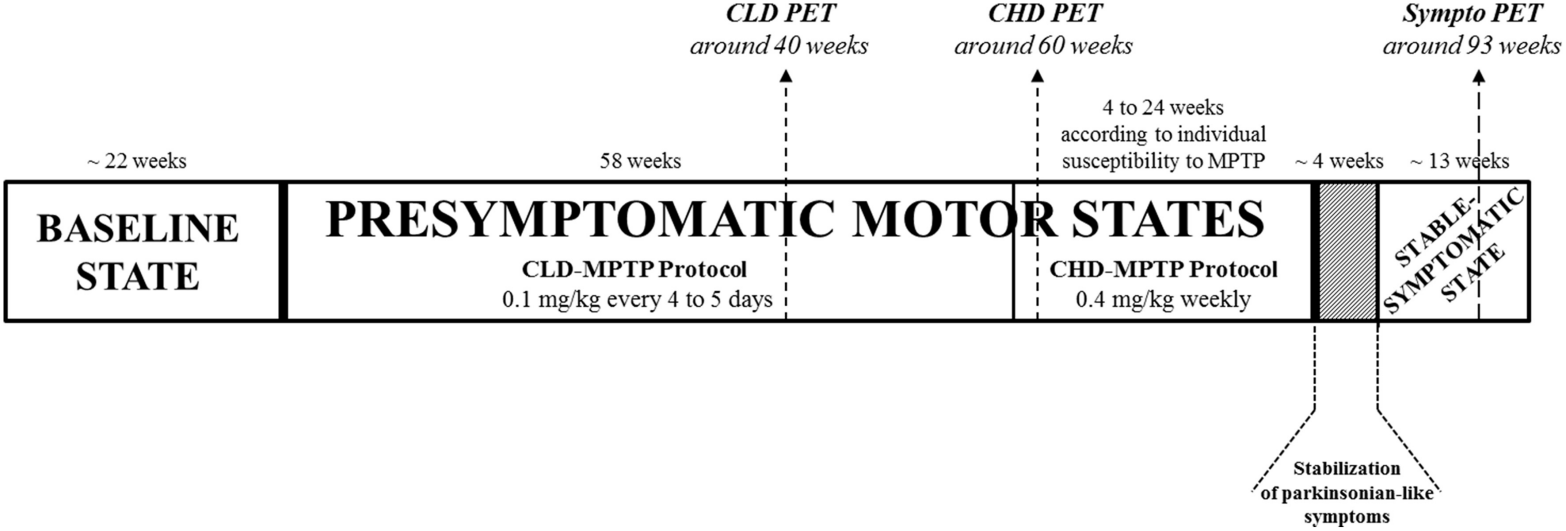
**Experimental design**. Animals had been previously trained for cognitive testing, and their social and motor behaviors were assessed over the 5 months preceding MPTP administration in order to have a global representation of the baseline state. Then, all animals (*n* = 6) were exposed to MPTP hydrochloride (MPTP-HCl) by systemic intramuscular (i.m.) administration. Firstly, animals received 0.1 mg/kg every 4–5 days for 58 weeks, i.e., chronic low dose CLD-MPTP protocol, to study the CLD-presymptomatic motor state. A second protocol was then used (6 days after the final CLD-MPTP injection), in which MPTP was injected once per week at 0.4 mg/kg under light anesthesia, i.e., chronic high-dose CHD-MPTP protocol, to study the stable-symptomatic motor state, although a short CHD-presymptomatic motor state had still been studied. During this second protocol, animals were administered 3–17 doses for a period of 4–24 weeks to develop the first parkinsonian-like motor symptoms, depending on individual body weight and the individual variability of sensitivity to MPTP. To stabilize the parkinsonian-like motor symptoms (≥1 month), animals received no or several additional MPTP injections according to fluctuations of the parkinsonian-like symptoms. One testing session was carried out at least 36 h after MPTP injection at 2–3 week intervals during the CLD-presymptomatic period and weekly during the CHD-presymptomatic and stable-symptomatic periods. These social, motor and cognitive evaluations were carried out over 4-day testing sessions (Day 1 = 1 h motor behavior assessment (2 animals) + cognitive testing, Day 2 = 1 h motor behavior assessment (2 animals) + 6 h social behavior assessment, Day 3 = 1 h motor behavior assessment (2 animals) + cognitive testing, Day 4 = 6 h social behavior assessment). Three PET scans were performed: the first 18 weeks before the end of the CLD-MPTP protocol (CLD PET), the second 2 weeks after CHD-MPTP protocol (CHD PET) and the third during the stable-symptomatic motor state (Sympto PET). All scanned animals were in the CLD-presymptomatic motor state during the CLD PET and also in the CHD-presymptomatic motor state during the CHD PET. All PET scans were performed at least 5 days after the MPTP injection.

All animals (*n* = 6) were exposed to MPTP hydrochloride (MPTP-HCl) (dissolved in saline, Sigma, St. Louis, USA) by systemic intramuscular (i.m.) administration. Firstly, animals received 0.1 mg/kg every 4–5 days for 58 weeks (10.7–12.3 mg/kg), i.e., chronic low dose CLD-MPTP protocol, to study the CLD-presymptomatic motor state. A second protocol was then used (6 days after the final CLD-MPTP injection), in which MPTP was injected once per week at 0.4 mg/kg under light anesthesia using ketamine (0.5 mg/kg) (Neumane et al., 2012), i.e., chronic high-dose CHD-MPTP protocol, to study the stable-symptomatic motor state, although a short CHD-presymptomatic motor state had still been studied. During this second protocol, animals were administered 3–17 doses for a period of 4–24 weeks (1.0–6.5 mg/kg) (Table 1; Figure 1).

**Table 1:** **Individual sensitivity to MPTP during the CLD-MPTP and the CHD-MPTP protocols (number of MPTP injections, total amount of MPTP administered (mg/kg) and related clinical score)**.

		**Dominant**			**Subordinate**		
		**Monkey A**	**Monkey B**	**Monkey C**	**Monkey D**	**Monkey E**	**Monkey F**
CLD-presymptomatic period	Number of injections during the CLD-MPTP protocol	110	110	110	110	110	110
	Total amount of MPTP (mg/kg) administered during the CLD-MPTP protocol	11.0	11.0	11.0	11.0	11.0	11.0
	Clinical score during the CLD-presymptomatic motor state	0.3	0.5	1.7	1	1.6	0.3
CHD-presymptomatic period	Number of MPTP injection during the CHD-MPTP protocol for the presymptomatic motor state	10	2	2	2	6	2
	Total amount of MPTP (mg/kg) administered during the CHD-MPTP protocol for the presymptomatic motor state	4.0	0.8	0.8	0.8	2.4	0.8
	Clinical score during the CHD-presymptomatic motor state	2.2	2.5	4.7	2.8	3.6	3.0
Stable-symptomatic period	Number of MPTP injection during the CHD-MPTP protocol for the stable-symptomatic motor state	7	3	3	1	1	1
	Total amount of MPTP (mg/kg) administered during the CHD-MPTP protocol for the stable-symptomatic motor state	2.8	1.2	1.2	0.4	0.4	0.4
	Clinic score during the stable-symptomatic motor state	6.5	7.4	7.6	7.9	8	7.6

### Experimental design

Animals had been previously trained for cognitive testing, and their social and motor behaviors were assessed over the 5 months preceding MPTP administration in order to have a global representation of the baseline state. One testing session was carried out at least 36 h after MPTP injection at 2–3 week intervals during the CLD-presymptomatic period and weekly during the CHD-presymptomatic and stable-symptomatic periods. Thus, 20 testing sessions were performed during the CLD-presymptomatic motor state. During the CHD-presymptomatic motor state, according to the animals, 2–10 testing sessions were performed and during the stable-symptomatic motor state 3 testing sessions were assessed. These social, motor and cognitive evaluations were carried out over 4-day testing sessions (Day 1 = 1 h motor behavior assessment (2 animals) + cognitive testing, Day 2 = 1 h motor behavior assessment (2 animals) + 6 h social behavior assessment, Day 3 = 1 h motor behavior assessment (2 animals) + cognitive testing, Day 4 = 6h social behavior assessment) (Figure 1).

### Behavioral study

No experimenters were present in the testing room [three 1.8 m^3^ cages (1 × 1 × 1.8 m) with separating grids removed to obtain an aviary system] during social and motor assessments. This was achieved through the use of three black and white wall cameras connected to a server and equipped with a Smart Digital Video Recorder for Life Sciences, also known as “Numeriscope” (View Point, Lyon, France). In addition, a color camera fixed to the ceiling and rotating 360° (View Point, Lyon, France) tracked animal movements in real-time and specifically zoomed in on the faces of the animals to discriminate different facial expressions. Each evaluation session was recorded to allow evaluations by two experimenters with no previous knowledge of the state of the animal.

#### Motor assessment

Video observation was used to evaluate animals, and motor behavior was scored five times during the 1-h protocol in order to have an average score for each motor test. Two animals, housed separately in adjacent cages (1 × 1 × 1.8 m for each cage), could be evaluated simultaneously and independently.

***Clinical rating scale.*** Clinical rating was inspired from the Canadian rating scale (Gomez-Mancilla et al., 1993; Chassain et al., 2001). Clinical symptoms data presented the total score for posture (0 = normal; 1 = intermittent flexion of trunk and limbs; 2 = constant flexion of trunk and limbs; 3 = crouch position), mobility (0 = normal; 1 = mild decrease; 2 = moderate decrease; 3 = severe decrease), gait (0 = normal; 1 = slow; 2 = very slow; 3 = very slow with freezing) and tremor (0 = absent; 1 = mild postural tremor; 2 = moderate postural tremor; 3 = resting tremor) (maximum total score of 12). A clinical score > 0 and = 6, was defined as the presymptomatic motor state; finally for a clinical score > 6, the individual was considered to be developing parkinsonian-like motor symptoms in stable-symptomatic motor state (Chassain et al., 2001).

***Locomotor activity assessment.*** Locomotor activity was assessed using a Vigie Primates® image analyzer system (View Point, Lyon, France) (Chassain et al., 2001). The system was comprised of a video camera connected to a video image analyzer system that calculated the quantity and quality of the locomotor activity in real time. The images were digitized with a 800 × 600 pixel definition on 256 gray levels, and the changes in gray level in pixels from one image to the next were counted every 80 ms to plot a raw activity curve (Chassain et al., 2001). It was possible to change the following parameters: (i) the detection sensitivity determining the threshold from which a pixel is considered to have changed from one image to the next, (ii) the duration of data acquisition before obtaining a summary of the activities of the animal in question during that period, and (iii) the duration of the experiment, which could range from one second to several days (a 1-h testing session during this study). From the raw curve, the activity of each animal could be separated into three states. The first state corresponded to inactivity of the animal, the second to normal activity and the third to hyperactivity. Thresholds between the three states could be adjusted to discriminate between the movements of the animal. During this study the time spent in an inactive state was specifically evaluated to ensure a good correlation between increased periods of inactivity and clinical motor disorders (Chassain et al., 2001).

#### Social assessment

***Dominance hierarchy.*** Hierarchical stability was verified over the 5 months preceding the first MPTP injection. It was measured via the tabulation of unidirectional conflicts and avoidances into a matrix of agonistic behaviors (aggressive and submissive behaviors) which were then reorganized into a dominance matrix (Angst, 1975; Bentley-Condit and Smith, 1999; Riddick et al., 2009).

Hierarchical status was assessed by the hierarchical steepness value, which shows the size of the absolute differences in overall dominance success between individuals of adjacent ranks. In other words, the steeper the slope between two individuals, the stronger the difference between two dominance ranks will be. The slope was measured using SOCPROG.6 software (Whitehead, 2009). Individual hierarchical status ranking was determined using the David's score for each animal (Gammell et al., 2003). The most dominant animal (monkey A) presented the highest David's score and the most subordinate animal (monkey F) presented the lowest value. The dominance hierarchy of the social group was linear and organized as followed: monkey A > monkey B > monkey C > monkey D > monkey E > monkey F.

**Figure 2:**
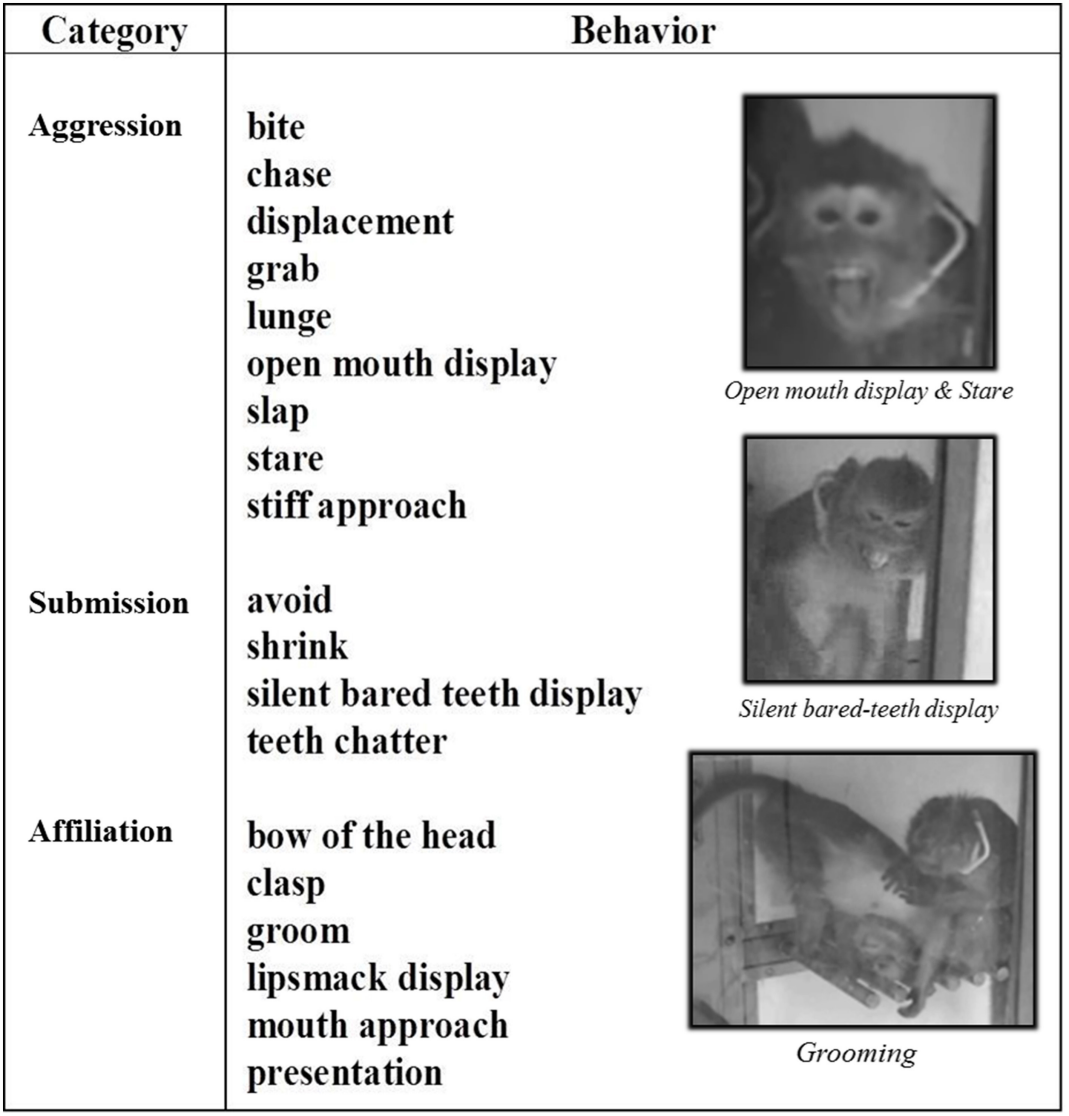
**Social behaviors of long-tailed macaques**. Social behaviors of long-tailed macaques are split into three categories: Aggression, Submission and Affiliation. In this study, 19 social behaviors were assessed, some of which required motor abilities (i.e., all facial expressions: open mouth display, stare, silent bared teeth display, teeth chatter, lipsmack display), and all of which required animal movement (displacement, stiff approach, lunge, chase, avoid).

***Social observations.*** In this study, 19 social behaviors were assessed and were categorized as aggressive, submissive or affiliative behaviors (Van Hooff, 1967; Morgan et al., 2000; Brent and Veira, 2002; Kaplan et al., 2002; Camus et al., 2013) (Figure 2). All behaviors required animal movement (displacement, stiff approach, lunge, chase, avoid) and some required facial expressions (open mouth display, stare, silent bared teeth display, teeth chatter, lipsmack display). For each animal, all social behaviors were recorded for 2 h using the focal animal sampling method (Altmann, 1974) and the LabWatcher software (View Point, Lyon, France). In these focal group sessions, each behavior was recorded in terms of emission and reception. The average frequency of the three categories of behavior over a period of 2 h was shown for each of the three motor states (normal, CLD-presymptomatic following CLD-MPTP protocol, CHD-presymptomatic following CHD-MPTP protocol and stable-symptomatic). The assessment order of each individual social behavior was randomly defined using the Kendall and BB Smith Table. All observations were carried out between 8.00 a.m. and 2.00 p.m. Animals were fed with fruit and vegetables at 7.30 am, and had *ad libitum* access to pellets and water during social behavior evaluation.

### Cognitive study: ORDT (object retrieval detour task)

The ORDT was used to assess the ability of the animals to retrieve an object (a piece of fruit) from inside a transparent box that only opened on one side (Taylor et al., 1990; Roeltgen and Schneider, 1991; Schneider and Pope-Coleman, 1995; Palfi et al., 1996; Vezoli et al., 2011). The Plexiglas box (15 × 15 × 5 cm) was fixed on a tray that was adapted to the home cage of the animal. The experimenter modified the cognitive level and motor skills required to solve the task and retrieve the piece of fruit by varying the location of the box in relation to the subject, the location of the reward inside the box, and finally the orientation of the open side of the box in relation to the subject. As shown in Supplementary Material, each testing session consisted of 35 trials that were randomly presented to the animals. This testing session contained “difficult” or “detour” trials in which the animal had to make a detour around a closed side of the box to reach the reward (detour to reach the reward: see configurations 1–20 in Supplementary Material), and “easy” trials which were defined as trials in which the opening of the box was facing the animal (no detour to reach the reward: see configurations 21–35 in Supplementary Material). Subjects were allowed 1 min to retrieve the reward, and then the box was set up for the next trial. The movements and responses of the animal involving the tray or the box were not restrained in any way.

Measures of performance included the number of successes and errors on detour or easy trials. Successes (retrieval of reward on the first reach) were expressed as a percentage of the total number of detour and easy trials. Errors (barrier hits, i.e., hitting a transparent side of the box) were expressed as percentage of responses observed (there could be several responses per trial except in the case of success) for easy or detour trials.

Four of the six animals were trained to come and work in the testing cage, without being forced in any way. Animals were trained during 11–18 sessions to achieve the cognitive task with a minimum of 75% successfully trials during three consecutive sessions. The other 2 animals (the third dominant animal: monkey C and the second subordinate animal: monkey E) refused to work on this test despite several weeks of training.

### Imaging study: PET scans

#### PET scan design

Owing to limited access to the PET facility, only 4 animals of the social group were selected. On the one hand, the 3 animals with the greatest behavioral social changes, namely the three most subordinate animals, were selected for the imaging part of the study, waiting for variation of ^18^F-DOPA uptake (Ki values) in the brain structures explored. On the other hand, the most dominant animal was also selected because of its essential role in the stability of the social group (Petit and Thierry, 1992). Three PET scans were performed: the first 18 weeks before the end of the CLD-MPTP protocol (CLD PET), the second 2 weeks after CHD-MPTP protocol (CHD PET) and the third during the stable-symptomatic motor state (Sympto PET). All scanned animals were in the CLD-presymptomatic motor state during the CLD PET and also in the CHD-presymptomatic motor state during the CHD PET. All PET scans were performed at least 5 days after the MPTP injection. Since no PET scan could be performed during the baseline state, all data obtained from the three PET scans for the four MPTP-treated animals were compared to data from PET scan performed in five randomly selected healthy animals (Control PET), considered as basal levels data. Finally, we compared the three PET scans performed for the four MPTP-treated animals.

It was important to note here that the measurement of ^18^F-DOPA uptake in the control healthy animas showed less than 10% variability, except in the subtantia nigra (13%), although no information was available regarding their hierarchical status. Moreover, previous PET studies with test-retest data have shown a good reproducibility of radiotracer binding measurement (Costes et al., 2007; Ballanger et al., 2013).

#### Acquisitions

Animals were fasted overnight prior to MRI (Magnetic Resonance Imaging) and PET exams. On the day of the experiment, animals were pretreated with Atropine (0.05 mg/kg i.m.) and were anesthetized with Zoletil (15 mg/kg i.m.) 15 min later. Lactated Ringer's solution was continuously infused through a saphenous vein catheter. Animals were then transported to the Imaging Center (CERMEP, Lyon, France) where they were placed on a stereotaxic apparatus. Respiratory frequency, pO2 and heart rate were monitored throughout the experiment. One hour after the anesthesia, PET scans were performed in a three-dimensional (3D) mode using a Siemens CTI HR+ tomograph, with an axial field of view of 15.2 cm, yielding 63 planes and a nominal in-plane resolution of 4.1 mm full width at half maximum (FWHM). Before the tracer injection, a transmission scan (68Ge rotating rod sources; 10 min) was acquired to correct for tissular 511 keV gamma attenuation. Dynamic acquisition started with the intravenous (i.v.) injection of ^18^F-DOPA (138 ± 7.4 MBq) (Neumane et al., 2012).

#### PET data processing and analysis

The 3D emission data were reconstructed with attenuation and scatter correction by a 3D filtered backprojection algorithm (Hamming filter; cut-off frequency, 0.5cycles/pixel) and a zoom factor of three, giving a transaxial resolution of 6.5 mm FWHM. Reconstructed volumes were 128 × 128 matrices of 0.32 × 0.32 mm^2^ pixels in sixty-three 2.42 mm spaced planes. The extraction of tissue time-activity concentration curves from automated delineated regions of interest was made possible by the specific *Macaca fascicularis* brain atlas (Ballanger et al., 2013). The influx rate constant of ^18^F-DOPA (Ki) was calculated by linearization of the graphical Patlak plot (Patlak et al., 1983) over 90 min post-injection using the cerebellum as the reference region.

During this study, a limited number of areas with significant ^18^F-DOPA uptake (Ki values) were assessed. Areas within the Basal Ganglia were the Anterior and Posterior Caudate Nucleus (Ant CdN/Post CdN), the Anterior Putamen (Ant Put), the ventral striatum (V Str), the Posterior Dorsal and Ventral Putamen (Post D Put/Post V Put) and the Substantia Nigra (SN). Areas assessed at the cortical level were the Insula (Ins), the Orbitofrontal Cortex (OFC), the Anterior Cingulate Cortex (ACC) and the Amygdala (Amyg). The cerebellum was the brain region reference.

### Statistical analysis

Statistical analyses were performed with Statistica statistical software (version 10; StatSoft, Inc. Tulsa, USA). After verifying that the data followed a normal distribution, we carried out Analysis of Variance with Repeated Measures followed by the Newman-Keuls *post-hoc* test for social, cognitive and locomotor activity data. Clinical motor score data were analyzed using a paired Student *t*-test. PET scans data analysis between groups were performed with Random Effects Models, making it possible to consider both the group as fixed effect and the intra-subject variability with subject as random effects. PET scans intra-group data analysis in the MPTP-treated animals were performed with Chi^2^-test. The correlations between measures were calculated using Pearson's correlation. In all cases, significance was accepted at the 95% of confidence level (*p* < 0.05).

## Results

### Motor behavior analyses

**Figure 3:**
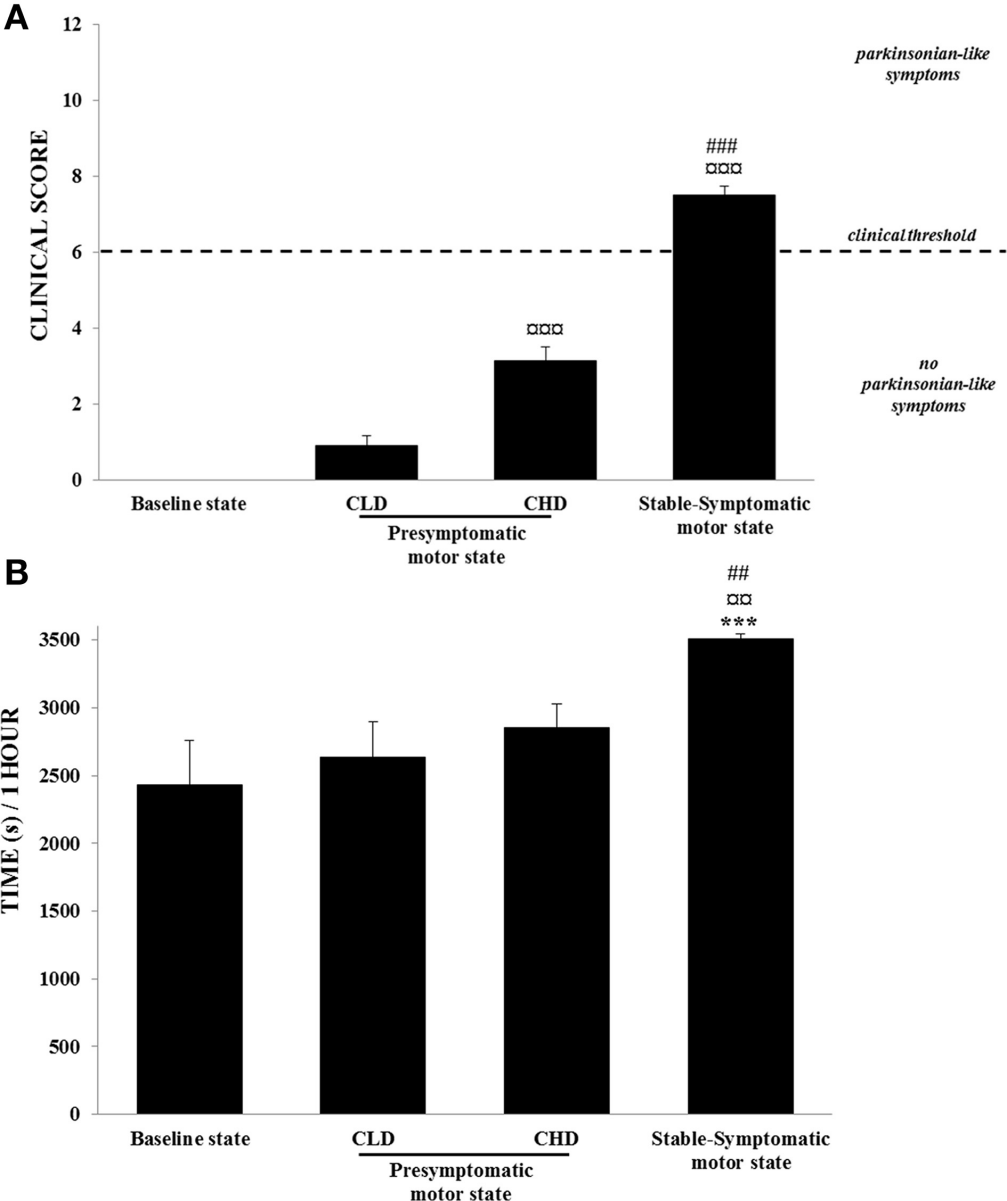
**Motor abilities from baseline to presymptomatic and symptomatic motor states (*n* = 6)**. (A) Clinical score: values were means ± SD (*n* = 6). ^¤¤¤^*p* < 0.001: CHD-presymptomatic and stable-symptomatic motor states vs. CLD-presymptomatic motor state; ^###^*p* < 0.001: stable-symptomatic motor state vs. CHD-presymptomatic motor state (Student *t*-test). **(B)** Time spent in inactivity: values were means ± SD (*n* = 6). ^***^p < 0.001: stable-symptomatic motor state vs. baseline state; ^¤¤^*p* < 0.01: symptomatic motor state vs. CLD-presymptomatic motor state; ^##^*p* < 0.01: stable-symptomatic motor state vs. CHD-presymptomatic motor state (ANOVA and *post-hoc* test).

No animals developed any parkinsonian-like motor symptoms during the CLD-MPTP protocol. The clinical score was below the threshold score of 6. No changes were observed in the time of inactivity from the baseline to the CLD-presymptomatic motor states (Figures 3A,B).

During the CHD-MPTP protocol following the CLD-MPTP protocol, two periods were observed. The first period corresponded to the CHD-presymptomatic motor state, and included a significant increase in the clinical score compared to the CLD-presymptomatic motor state (3.1 ± 0.9 vs. 0.9 ± 0.6; *t* = 11.28, *p* < 0.001), although the score stayed below the threshold value of 6. No significant increase in the time of inactivity was observed during this state. The second period corresponded to the appearance of stable parkinsonian-like motor symptoms, and a significant increase of clinical score was observed in comparison to the CHD-presymptomatic (7.5 ± 0.6 vs. 3.1 ± 0.9; *t* = 13.26, *p* < 0.001) and the CLD-presymptomatic (0.9 ± 0.6; *t* = 29.97, *p* < 0.001) motor states. At this state, all animals had a clinical score > 6. The time spent in inactivity during this period was positively correlated with clinical score (*r* = 0.69, *p* < 0.001) and was significantly higher than the CHD-presymptomatic [3510.9 ± 81.1 s vs. 2853.6 ± 435.0 s; *F*_(3, 20)_ = 9.69, *p* < 0.001; *post-hoc* test, *p* < 0.01] and the CLD-presymptomatic [2633.2 ± 644.5 s; *F*_(3, 20)_ = 9.69, *p* < 0.001; *post-hoc* test, *p* < 0.01] motor states (Figures 3A,B). No significant difference was observed between the two subgroups (Dominant vs. Subordinate). However, high individual variability was observed in the sensitivity to MPTP, with varying numbers of CHD-MPTP injections and amounts of MPTP required to develop parkinsonian-like motor symptoms. Indeed, monkeys B, C, D, and F seemed to be more sensitive to MPTP than monkeys A and E (Table 1).

### Social analyses

#### Baseline state

**Figure 4:**
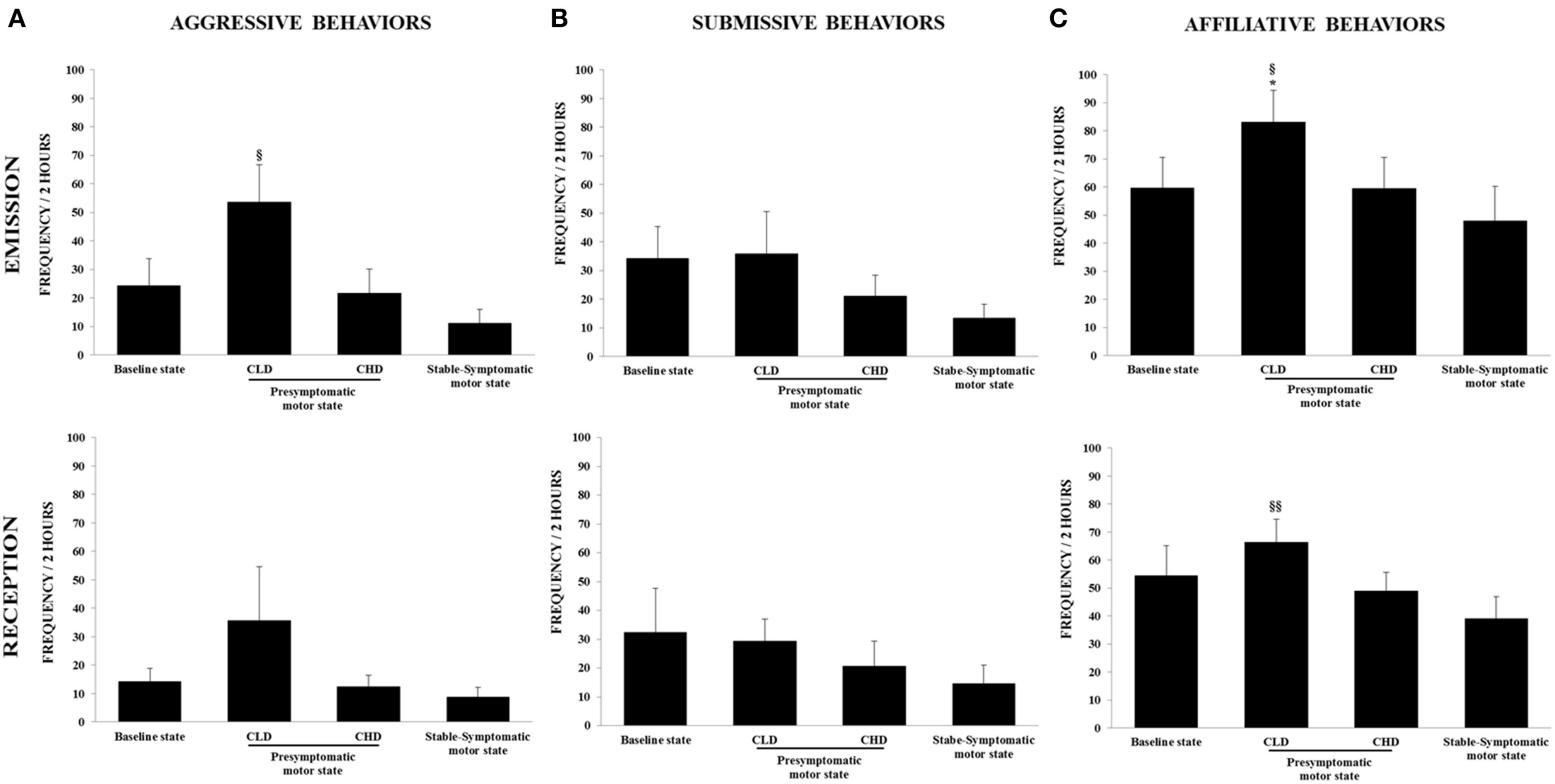
**Evolution of social behaviors from baseline to presymptomatic and symptomatic motor states within the social group (*n* = 6). (A)** Aggressive behaviors were recorded in terms of emission and reception using the focal animal sampling method for each monkey. Values were means ± SD (*n* = 6). ^§^*p* < 0.05: CLD-presymptomatic motor state vs. stable-symptomatic motor state (ANOVA and *post-hoc* test). **(B)** Submissive behaviors were recorded in terms of emission and reception using the focal animal sampling method for each monkey. **(C)** Affiliative behaviors were recorded in terms of emission and reception using the focal animal sampling method for each monkey. Values were means ± SD (*n* = 6). ^*^p < 0.05: CLD-presymptomatic motor state vs. baseline state; ^§§^ *p* < 0.01^§^ *p* < 0.05: CLD-presymptomatic motor state vs. stable-symptomatic motor state (ANOVA and *post-hoc* test).

The baseline frequency of agonistic behaviors, i.e., aggressive behaviors (emitted: 24.2 ± 23.3; received: 14.2 ± 11.3) and submissive behaviors (emitted: 34.1 ± 27.2; received: 32.3 ± 37.8), reflected the formal dominance within the social group, i.e., each animal recognized its own social status and that of its congeners (Figures 4A,B). The affiliative behaviors category showed the highest frequency of appearance (emitted: 59.7 ± 26.7; received: 54.3 ± 26.2) (Figure 4C).

#### CLD-presymptomatic motor state

There was no significant change in the frequency of emitted and received aggressive behaviors from the CLD-presymptomatic motor state compared to the baseline state, although a marked increase was observed (emitted: 53.5 ± 32.2 vs. 24.2 ± 23.3; received: 35.7 ± 46.2 vs. 14.2 ± 11.3) (Figure 4A). No significant changes were seen in the frequency of emitted and received submissive behaviors (Figure 4B). The frequency of emitted affiliative behaviors significantly increased during the CLD-presymptomatic motor state compared to the baseline state [83.1 ± 27.7 vs. 59.7 ± 26.7; *F*_(3, 20)_ = 4.02, *p* < 0.05; *post-hoc* test, *p* < 0.05] (Figure 4C).

**Figure 5:**
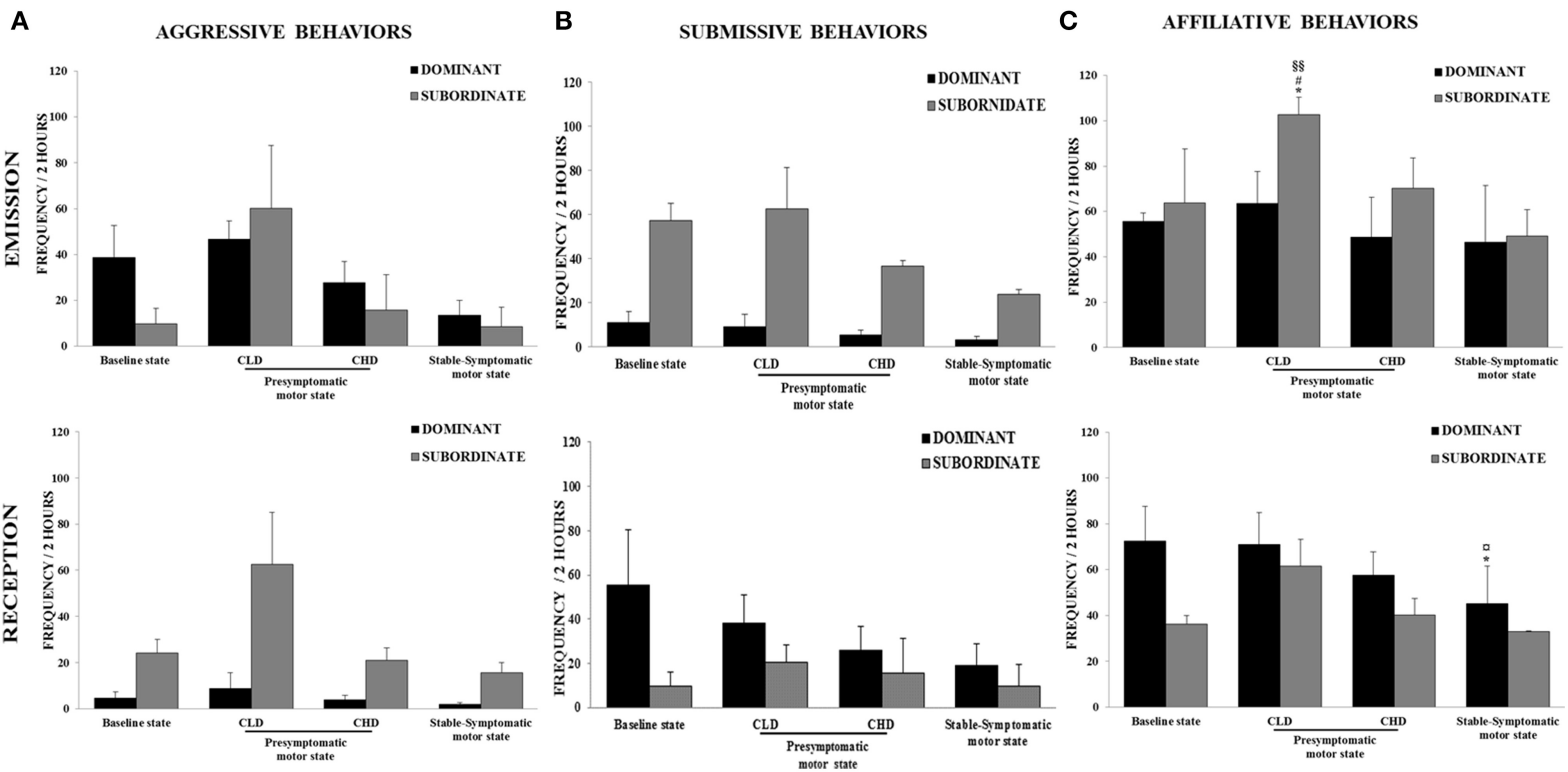
**Evolution of social behaviors from baseline to presymptomatic and symptomatic motor states within the two social subgroups (*n* = 3 in each subgroup). (A)** Aggressive behaviors were recorded in terms of emission and reception using the focal animal sampling method for each monkey. **(B)** Submissive behaviors were recorded in terms of emission and reception using the focal animal sampling method for each monkey. **(C)** Affiliative behaviors were recorded in terms of emission and reception using the focal animal sampling method for each monkey. Values were means ± SD (Dominant group: *n* = 3; Subordinate group: *n* = 3). ^*^p < 0.05: Subordinate CLD-presymptomatic motor state vs. baseline state; ^#^*p* < 0.05: Subordinate CLD-presymptomatic motor state vs. CHD-presymptomatic motor state; ^§§^*p* < 0.01: Subordinate CLD-presymptomatic motor state vs. stable-symptomatic motor state; ^*^p < 0.05: Dominant stable-symptomatic motor state vs. baseline state; ^¤^*p* < 0.05: Dominant stable-symptomatic motor state vs. CLD-presymptomatic motor state (ANOVA and *post-hoc* test).

In subgroup social analyses, there was no significant change in the frequency of emitted and received aggressive behaviors. However, a marked increase in the frequency of emitted and received aggressive behaviors in subordinate animals was observed during the CLD-presymptomatic motor state compared to the baseline state (emitted: 60.2 ± 47.6 vs. 9.8 ± 11.6; received: 62.6 ± 55.0 vs. 24.0 ± 3.1) (Figure 5A). No significant changes were seen in the frequency of emitted and received submissive behaviors whatever the social subgroup (Figure 5B). No change occurred in the frequency of emitted affiliative behaviors of dominant animals. Unlike dominant animals, the frequency of emitted affiliative behaviors of subordinate animals significantly increased during the CLD-presymptomatic motor state compared to the baseline state [102.6 ± 13.4 vs. 63.8 ± 41.1; *F*_(3, 8)_ = 9.43, *p* < 0.05; *post-hoc* test, *p* < 0.05]. No significant change occurred in the frequency of received affiliative behaviors of dominant and subordinate animals (Figure 5C).

Individual data (Table 2) were used to determine the inter-individual involvement of each animal in these marked changes observed during the CLD-presymptomatic motor state compared to the baseline state. Monkey A, the most dominant animal, presented no major variation in its frequency of emitted aggressive behaviors in terms of emission or reception. Moreover, whilst its frequency of emitted affiliative behaviors increased almost two-fold, no variation was noted in its frequency of received affiliative behaviors. Monkey B, the second most dominant animal, presented no major variation in its frequency of emitted aggressive behaviors but the reception of aggressive behaviors was half as high as the baseline values. Moreover, it did not present any major variation in its frequency of affiliative behaviors in terms of emission or reception. The four others animals in the social group showed a higher variation in the frequency of aggressive behaviors. Indeed, the frequency of emitted aggressive behaviors in monkey C increased almost three-fold, whilst its frequency of received aggressive behaviors increased six-fold. Moreover, it did not show any major variation of its frequency of affiliative behaviors, in terms of emission and reception. For monkey D, its frequency of emitted aggressive behaviors decreased about 3.5 times whilst the frequency of received aggressive behaviors increased almost five-fold. Moreover its frequency of emitted affiliative behaviors increased almost two-fold and no major variation was observed in its frequency of received affiliative behaviors. Monkey E presented a marked increase of about 14.5 times its frequency of emitted aggressive behaviors and did not show any variation in its frequency of received aggressive behaviors. Moreover, it did not shown any major variation in its frequency of emitted affiliative behaviors but its frequency of received affiliative behaviors increased almost two-fold. Finally, the most subordinate animal, monkey F, surprisingly emitted aggressive behaviors that had not been observed during the baseline state, and did not show any major variation in its frequency of received aggressive behaviors. Moreover, it showed a three-fold increase in its frequency of emitted affiliative behaviors and its frequency of received affiliative behaviors increased almost two-fold.

**Table 2:** **Individual frequency of the three categories of social behaviors during the baseline state, the CLD and CHD-presymptomatic motor states and the stable-symptomatic motor state**.

		**Dominant**			**Subordinate**		
		**Monkey A**	**Monkey B**	**Monkey C**	**Monkey D**	**Monkey E**	**Monkey F**
Frequency of EMITTED AGGRESSIVE BEHAVIORS	Baseline state	58.0	46.7	11.3	22.7	6.7	0.0
	CLD-presymptomatic motor state	47.8	60.0	32.5	6.5	97.0	77.2
	CHD-presymptomatic motor state	17.1	46.0	19.7	46.5	0.6	0.0
	Stable-symptomatic motor state	26.3	6.0	8.3	25.3	0.3	0.0
Frequency of RECEIVED AGGRESSIVE BEHAVIORS	Baseline state	0.0	9.3	4.0	24.7	26.7	20.7
	CLD-presymptomatic motor state	0.1	4.2	22.0	126.1	28.5	33.3
	CHD-presymptomatic motor state	0.1	7.0	4.3	21.5	17.0	24.5
	Stable-symptomatic motor state	0.0	2.7	2.7	15	11.3	20.7
Frequency of EMITTED SUBMISSIVE BEHAVIORS	Baseline state	1.3	17.3	14.7	44.7	72.0	54.7
	CLD-presymptomatic motor state	0.6	6.7	19.7	99.3	39.1	49.3
	CHD-presymptomatic motor state	1.1	8.0	7.3	38.0	32.1	40.0
	Stable-symptomatic motor state	0.0	4.7	4.7	20.7	22.0	28.3
Frequency of RECEIVED SUBMISSIVE BEHAVIORS	Baseline state	96.7	58.7	10.0	22.7	5.3	0.7
	CLD-presymptomatic motor state	52.8	48.6	12.4	8.9	35.3	17.5
	CHD-presymptomatic motor state	21.9	46.0	9.3	46.5	0.4	0.0
	Stable-symptomatic motor state	38.3	10.7	8.7	29.3	0.0	0.0
Frequency of EMITTED AFFILIATIVE BEHAVIORS	Baseline state	49.3	62.7	54.7	54.7	108.7	28.0
	CLD-presymptomatic motor state	87.6	64.2	38.9	99.7	117.3	91.0
	CHD-presymptomatic motor state	84.0	30.5	31.3	76.0	90.1	44.5
	Stable-symptomatic motor state	96.3	22.7	20.3	49.0	69.7	29.0
Frequency of RECEIVED AFFILIATIVE BEHAVIORS	Baseline state	101.3	66.0	50.0	43.3	30.7	34.7
	CLD-presymptomatic motor state	90.5	78.7	43.8	46.1	54.5	84.3
	CHD-presymptomatic motor state	77.4	52.5	42.7	51.5	27.5	42.0
	Stable-symptomatic motor state	78.0	30.7	26.7	33.0	33.3	33.0

Interestingly, the dominance hierarchy within the social group was modified during the CLD-presymptomatic motor state (A>B>C>E>F>D) compared to the baseline state (A>B>C>D>E>F). Thus, monkey D presented the lowest hierarchical status.

#### CHD-presymptomatic motor state

There was no significant change in the frequency of emitted and received aggressive behaviors compared to the baseline and the CLD-presymptomatic motor states, although a marked decrease was observed during the CHD-presymptomatic motor state compared to the CLD-presymptomatic motor state (emitted: 21.7 ± 20.7 vs. 53.5 ± 32.2; received: 12.4 ± 10.0 vs. 35.7 ± 46.2) (Figure 4A). No significant changes were seen in the frequency of emitted and received submissive behaviors. However, a decrease in the frequency of emitted submissive was observed during the CHD-presymptomatic motor state compared to the CLD-presymptomatic motor state (21.1 ± 17.5 vs. 35.8 ± 36.3) (Figure 4B). There was no significant change in the frequency of emitted and received affiliative behaviors compared to the baseline and the CLD-presymptomatic motor states, although a decrease was observed during the CHD-presymptomatic motor state compared to the CLD-presymptomatic motor state (emitted: 59.4 ± 27.1 vs. 83.1 ± 27.7; received: 48.9 ± 16.6 vs. 66.3 ± 20.6) (Figure 4C).

In subgroup social analyses, there was no significant change in the frequency of emitted and received aggressive behaviors. However, a marked decrease in the frequency of emitted and received aggressive behaviors in subordinate animals was observed during the CHD-presymptomatic motor state compared to CLD-presymptomatic motor state (emitted: 15.7 ± 26.7 vs. 60.2 ± 47.6; received: 21.0 ± 3.8 vs. 62.6 ± 55.0). No significant change in the frequency or emitted and receive submissive behaviors was observed. However, a marked decrease in the frequency of emitted submissive behaviors in subordinate animals was observed during the CHD-presymptomatic motor state compared to the CLD-presymptomatic motor state (36.7 ± 4.1 vs. 62.6 ± 32.3). No significant change in the frequency or emitted and receive affiliative behaviors was observed. However, a marked decrease in the frequency of emitted affiliative behaviors in subordinate animals was observed during the CHD-presymptomatic motor state compared to the CLD-presymptomatic motor state (70.2 ± 23.4 vs. 102.6 ± 13.4).

Individual data (Table 2) were used to determine the inter-individual involvement of each animal in these marked changes observed during the CHD-presymptomatic motor state compared to the CLD-presymptomatic motor state. Monkey A, the most dominant animal, presented a decrease about 3 times in its frequency of emitted aggressive behaviors and did not show any variation in its frequency of received aggressive behaviors. No variation in its frequency of emitted submissive behaviors was observed, whilst its frequency of received submissive behaviors decreased almost two-fold. Finally, monkey A did not present any variation in its frequency of affiliative behaviors in terms of emission and reception. Monkey B, the second most dominant animal, presented no major variation in its frequency of aggressive and submissive behaviors in terms of emission and reception. Moreover, its frequency of emitted affiliative behaviors decreased almost two-fold, whilst no major variation in its frequency of received affiliative behaviors was observed. The four others animals in the social group showed a higher variation in the frequency of social behaviors. Indeed, the frequency of emitted aggressive behaviors in monkey C decreased almost two-fold, whilst its frequency of received aggressive behaviors decreased about 5 times. Its frequency of emitted submissive behaviors decreased almost three-fold whilst no major variation in its frequency of received submissive behaviors was observed. Finally, monkey C did not present any variation in its frequency of affiliative behaviors in terms of emission and reception. For monkey D, its frequency of emitted aggressive behaviors increased about 7 times and its frequency of received aggressive behaviors decreased almost six-fold. Its frequency of emitted submissive behaviors decreased almost three-fold, whilst its frequency of received submissive behaviors increased about 5 times. Finally, monkey D did not present any variation in its frequency of affiliative behaviors in terms of emission and reception. Monkey E presented a marked decrease of about 162 times in its frequency of emitted aggressive behaviors and did not show any variation in its frequency of received aggressive behaviors. Its frequency of emitted submissive behaviors did not present any variation, whilst its frequency of received submissive behaviors decreased about 88 times. Finally, monkey E did not present any variation in its frequency of emitted affiliative behaviors, whilst its frequency of received affiliative behaviors decreased almost two-fold. The most subordinate animal, monkey F presented no emission of aggressive behaviors during the CHD-presymptomatic motor state as observed during the baseline state and did not show any major variation in its frequency of received aggressive behaviors. Moreover, its frequency of emitted submissive behaviors did not present any variation, whilst any reception of submissive behaviors was observed as during the baseline state. Finally, monkey F presented a decrease in its frequencies of emitted and received affiliative behaviors almost two-fold.

The dominance hierarchy within the social group was modified during the CHD-presymptomatic motor state (A>B>C>D>E>F) compared to the CLD-presymptomatic motor state (A>B>C>E>F>D). Thus, monkey D recovered its initial hierarchical status.

#### Stable-symptomatic motor state

A significant difference was observed for the emitted aggressive behaviors during the stable-symptomatic motor state compared to the CLD-presymptomatic motor state [53.5 ± 32.2 vs. 11.1 ± 11. 9; *F*_(3, 20)_ = 3.27, *p* = 0.05; *post-hoc* test, *p* < 0.05] but not the CHD-presymptomatic motor state and the baseline state (Figure 4A). No significant changes were seen in the frequency of emitted and received submissive behaviors, although these frequencies seemed to be lower during the stable-symptomatic motor state than during the three others states (Figure 4B). A significant difference was observed for the emitted affiliative behaviors during the stable-symptomatic motor state compared to the CLD-presymptomatic motor state [83.1 ± 27.7 vs. 47.8 ± 30.3; *F*_(3, 20)_ = 4.02, *p* < 0.05; *post-hoc* test, *p* < 0.05] but not the CHD-presymptomatic motor state and the baseline state. The frequency of received affiliative behaviors also significantly decreased during the stable-symptomatic motor state compared to the CLD-presymptomatic motor state [39.1 ± 19.2 vs. 66.3 ± 20.6; *F*_(3, 20)_ = 5.75, *p* < 0.01; *post-hoc* test, *p* < 0.01] (Figure 4C).

In subgroup social analyses, there was no significant changes in the frequency of emitted and received aggressive behaviors during the stable-symptomatic motor state compared to the three others states. However, the frequency of emitted aggressive behaviors in dominant animals seemed to be lower during the stable-symptomatic motor state than during the three others states. In subordinate animals, the frequency of aggressive behaviors in terms of emission and reception seemed to be lower during the stable-symptomatic motor state compared to the CLD-presymptomatic motor state (emitted: 8.6 ± 14.5 vs. 60.2 ± 47.6; received: 15.7 ± 4.7 vs. 62.6 ± 55.0) but not the CHD-presymptomatic motor state and the baseline state (Figure 5A). No significant changes were seen in the frequency of emitted and received submissive behaviors in subgroup social analyses. However, the frequency of emitted submissive behaviors in subordinate animals seemed to be lower during the stable-symptomatic motor state than during the three others states (Figure 5B). No change occurred in the frequency of emitted affiliative behaviors of dominant animals whereas the frequency of received affiliative behaviors was significantly lower during the stable-symptomatic motor state compared to the baseline [45.11 ± 28.55 vs. 72.44 ± 26.27; *F*_(3, 8)_ = 6.33, *p* < 0.05; *post-hoc* test, *p* < 0.05] and the CLD-presymptomatic motor states [71.0 ± 24.3; *F*_(3, 8)_ = 6.33, *p* < 0.05; *post-hoc* test, *p* < 0.05]. In subordinate animals, the frequency of affiliative behaviors in terms of emission and reception seemed to be lower during the stable-symptomatic motor state compared to the CLD-presymptomatic motor state (emitted: 49.2 ± 20.3 vs. 102.6 ± 13.4; received: 33.1 ± 0.2 vs. 61.6 ± 20.1) but not the CHD-presymptomatic motor state and the baseline state (Figure 5C).

Individual data (Table 2) showed that a global decrease was observed in the frequency of emission and reception of all social behaviors during the stable-symptomatic motor state compared to the CHD-presymptomatic motor state, except for the most dominant animal. Moreover, any variation of the dominance hierarchy within the social group was observed during the stable-symptomatic motor state compared to the CHD-presymptomatic motor state. Thus, the dominance hierarchy was similar to the dominance hierarchy during the baseline state.

#### Correlation analyses

A positive correlation was observed between the frequency of emitted aggressive behaviors and the frequency of received submissive behaviors (*r* = 0.69, *p* < 0.001). A positive correlation was observed between the frequency of emitted aggressive behaviors and the frequency of received affiliative behaviors (*r* = 0.66, *p* < 0.001). A positive correlation was observed between the frequency of received aggressive behaviors and the frequency of emitted submissive behaviors (*r* = 0.86, *p* < 0.001). A negative correlation was observed between the frequency of emitted submissive behaviors and the frequency of received submissive behaviors (*r* = −0.41, *p* < 0.05). A positive correlation was observed between the frequency of received submissive behaviors and the frequency of received affiliative behaviors (*r* = 0.77, *p* < 0.001). Finally, a negative correlation was observed between the time spent in inactivity and the frequency of emitted aggressive behaviors (*r* = −0.51, *p* < 0.05).

### ORDT performance

**Figure 6:**
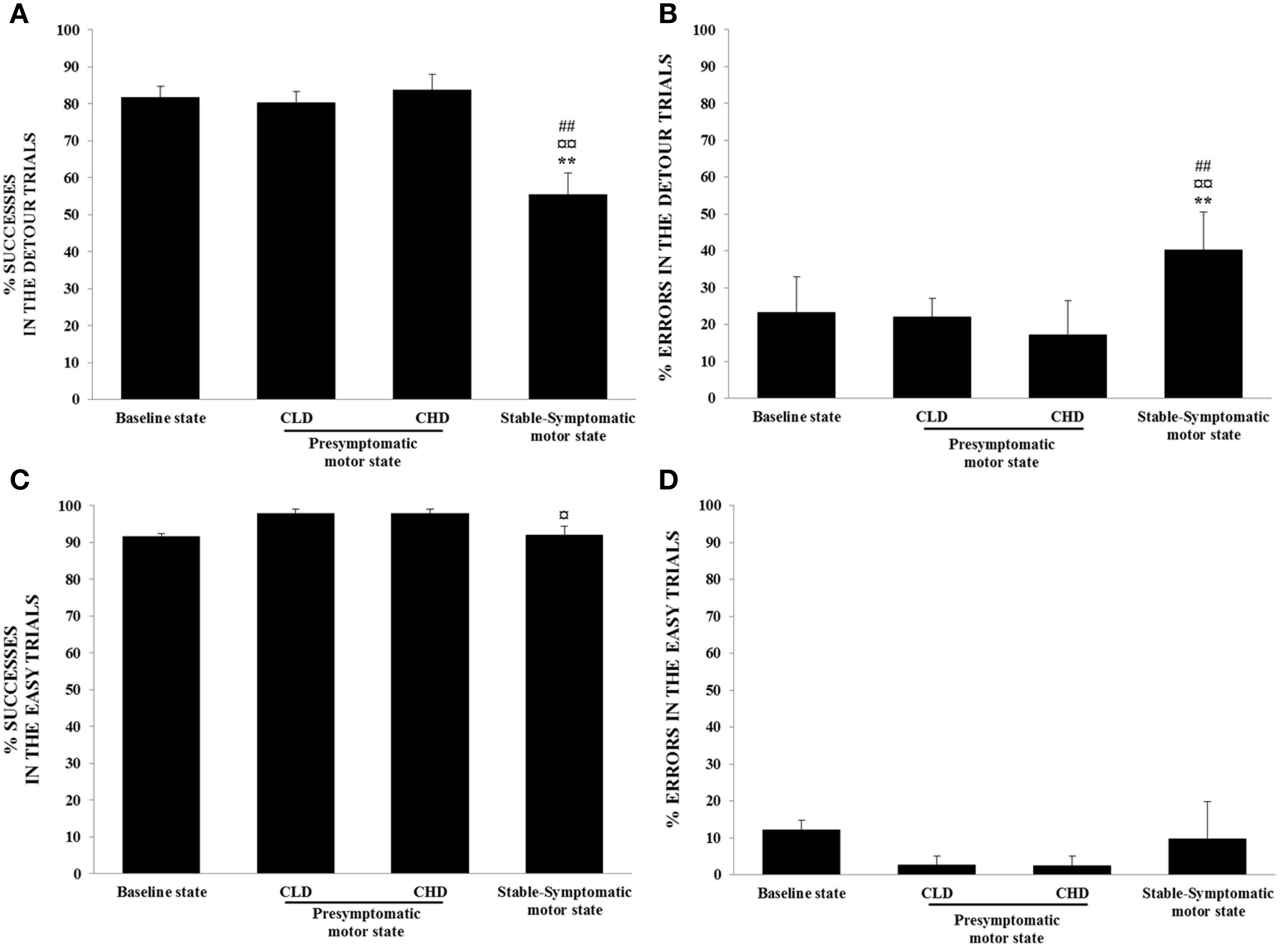
**Evolution of ORDT performance from baseline to presymptomatic and symptomatic motor states (*n* = 4). (A)** Successes in the detour trials: successes (retrieval of reward on the first reach) were expressed as percentage of total number of detour trials. Values were means ± SD (*n* = 4). ^**^p < 0.01: stable-symptomatic motor state vs. baseline state; ^¤¤^*p* < 0.01: stable-symptomatic motor state vs. CLD-presymptomatic motor state; ^##^*p* < 0.01: stable-symptomatic motor state vs. CHD-presymptomatic motor state (ANOVA and *post-hoc* test). **(B)** Errors in the detour trials: errors (barrier hits, i.e., hitting a transparent side of the box) were expressed as a percentage of responses observed for detour trials. Values were means ± SD (*n* = 4). ^**^p < 0.01: stable-symptomatic motor state vs. baseline state; ^¤¤^*p* < 0.01: stable-symptomatic motor state vs. CLD-presymptomatic motor state; ^##^*p* < 0.01: stable-symptomatic motor state vs. CHD-presymptomatic motor state (ANOVA and *post-hoc* test). **(C)** Successes in the easy trials: successes (retrieval of reward on the first reach) were expressed as a percentage of the total number of easy trials. Values were means ± SD (*n* = 4). ^¤^*p* < 0.05: stable-symptomatic motor state vs. CLD-presymptomatic motor state (ANOVA and *post-hoc* test). **(D)** Errors in the easy trials: errors (barrier hits, i.e., hitting a transparent side of the box) were expressed as a percentage of responses observed for easy trials.

The success rate for detour trials was negatively correlated with the clinical scores of animals (*r* = −0.80, *p* < 0.001) and significantly decreased during the stable-symptomatic state compared to the baseline [55.4 ± 11.8% vs. 81.7 ± 5.9%; *F*_(3, 12)_ = 10.52, *p* < 0.01; *post-hoc* test, *p* < 0.01], the CLD-presymptomatic motor [80.4 ± 6.0%; *F*_(3, 12)_ = 10.52, *p* < 0.01; *post-hoc* test, *p* < 0.01] and the CHD-presymptomatic motor states [83.7 ± 8.4%; *F*_(3, 12)_ = 10.52, *p* < 0.01; *post-hoc* test, *p* < 0.01] (Figure 6A). The percentage of errors responses observed in the detour trials was positively correlated with the time spent in inactivity (*r* = 0.60, *p* < 0.01) and significantly increased during the stable-symptomatic state compared to the baseline [40.3 ± 10.3% vs. 23.2 ± 9.8%; *F*_(3, 12)_ = 9.83, *p* < 0.01; *post-hoc* test, *p* < 0.01], the CLD-presymptomatic motor [22.1 ± 5.0%; *F*_(3, 12)_ = 9.83, *p* < 0.01; *post-hoc* test, *p* < 0.01] and the CHD-presymptomatic motor states [17.2 ± 9.3%; *F*_(3, 12)_ = 9.83, *p* < 0.01; *post-hoc* test, *p* < 0.01] (Figure 6B). Slight changes were seen in the percentage of successes in the easy trials, with a significant decrease in values during the stable-symptomatic motor state compared to the CLD-presymptomatic motor state [92.1 ± 4.7% vs. 97.8 ± 2.6%; *F*_(3, 12)_ = 4.52, *p* < 0.05; *post-hoc* test, *p* < 0.05] (Figure 6C). Any significant differences between the four states (baseline state and the three motor states) was observed for the percentage of errors in the easy trials (Figure 6D). No significant difference was observed between the two subgroups (Dominant vs. Subordinate).

A negative correlation between the success rate for detour trials and the percentage of errors responses in the detour trials was observed (*r* = 0.92, *p* < 0.001). Similarly, a negative correlation was observed between the success rate for easy trials and the percentage of errors responses in the easy trials (*r* = 0.93, *p* < 0.001).

Furthermore, the percentage of errors responses in the detour trials was negatively correlated with the frequency of received affiliative behaviors (*r* = −0.52, *p* < 0.05).

### ^18^F-DOPA uptake

#### Comparisons with the control group

**Figure 7:**
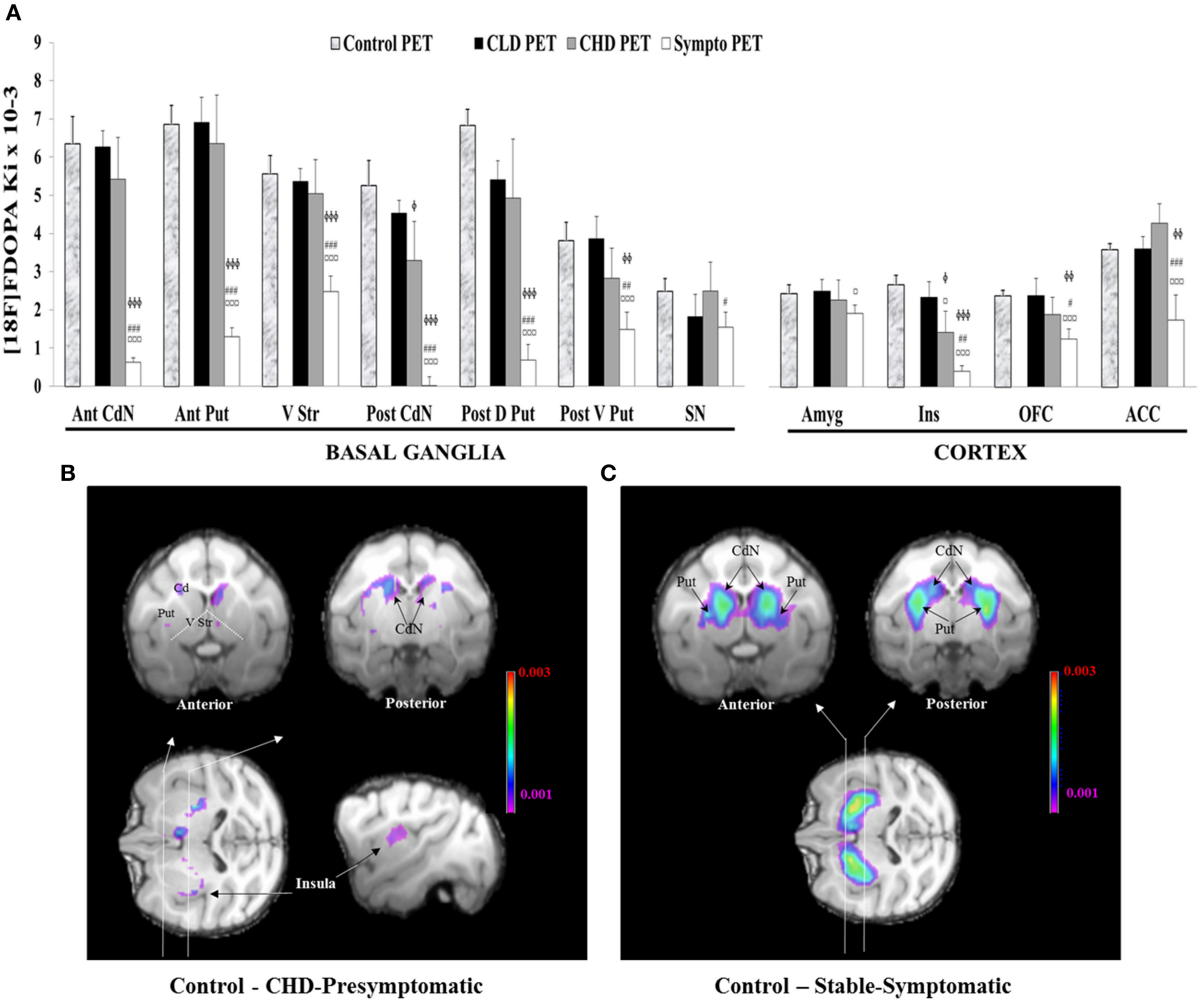
**Comparisons of ^18^F-DOPA uptake in PET imaging. (A)** The rate of specific uptake (Ki values) was assessed within basal ganglia in relation to ^18^F-DOPA uptake in the reference brain region (cerebellum). The basal ganglia includes the Anterior and Posterior Caudate Nucleus (Ant CdN/Post CdN), the Anterior Putamen (Ant Put), the Posterior Dorsal and Ventral Putamen (Post D/V Put), the Ventral Striatum (V Str) and the Substantia Nigra (SN), as well as uptake in cortical regions such as the Anterior Cingulate Cortex (ACC), the Orbitofrontal Cortex (OFC), the Amygdala (Amyg) and the Insula (Ins). One PET scan was carried out for the control group (*n* = 5) and was considered the Control PET. Three PET scans were carried out for four animals of the social group (*n* = 4) of the study: the first at the end of the presymptomatic motor state following CLD-MPTP protocol (CLD PET), the second during the presymptomatic motor state following CHD-MPTP protocol (CHD PET) period and the third during the symptomatic motor state, when animals developed stable parkinsonian-like motor symptoms (≥1 month) (Sympto PET). Comparison with the control group: values of the Control PET scan were means ± SD (*n* = 5). Values of the three other PET scans were means ± SD (*n* = 4). ^ϕϕϕ^*p* < 0.001, ^ϕϕ^*p* < 0.01, ^ϕ^*p* < 0.05; CHD PET, Sympto PET vs. Control PET (random effects models). Comparison within the social group: values of the three PET scans were means ± SD (*n* = 4). ^¤¤¤^*p* < 0.001, ^¤^*p* < 0.05: CHD PET, Sympto PET vs. CLD PET; ^###^*p* < 0.001, ^##^*p* < 0.01, ^#^*p* < 0.05: Sympto PET vs. CHD PET (Chi^2^-test). **(B)** Subtraction images between the average ^18^F-DOPA uptake of control PET and the average ^18^F-DOPA uptake of CHD PET. ^18^F-DOPA uptake decreases in the Post CdN and the insula were illustrated in frontal sections (black arrows). **(C)** Subtraction images between the average ^18^F-DOPA uptake of control PET and the average ^18^F-DOPA uptake of sympto PET.

The comparison of ^18^F-DOPA uptake (Ki value) between the control PET and the CLD PET showed no significant difference, whatever the cerebral area concerned (Figure 7A).

^18^F-DOPA uptake between the control PET and the CHD PET significantly decreased in the Post CdN (3.3 ± 2.3 vs. 5.3 ± 1.5; random effect models, *z* = 2.20, *p* < 0.05) and the Ins (1.4 ± 1.1 vs. 2.7 ± 0.6; random effect models, *z* = 2.55, *p* < 0.05). These results were intensified during the sympto PET compared to the control PET (Post CdN: 0 ± 0.5 vs. 5.3 ± 1.5; random effect models, *z* = 5.89, *p* < 0.001; Ins: 0.4 ± 0.3 vs. 2.7 ± 0.6; random effect models, *z* = 4.67, *p* < 0.001) (Figures 7A,B).

During the sympto PET, most regions of the basal ganglia and the cortex showed a significant decrease in ^18^F-DOPA uptake compared to the control PET. However, a greater decrease was observed in ^18^F-DOPA uptake in Ant / Post CdN (around 95%: 94.7 ± 5.7) than in Ant / Post Put (around 80%: 80.2 ± 10.5), the V Str (around 55%: 55.3 ± 14.6) and the SN (around 38%: 37.6 ± 31.6). Similarly, a greater decrease in ^18^F-DOPA uptake was observed in the Ins (around 85%: 84.8 ± 11.4) than in the OFC (around 47%: 47.2 ± 20.6) and the ACC (around 50%: 51.4 ± 37.0). ^18^F-DOPA uptake in the Amyg did not significantly decrease, even during the symptomatic state (Figures 7A,C).

#### Comparisons within the social group

The only significant decrease in ^18^F-DOPA uptake during the CHD PET compared to the CLD PET was observed in the Ins (1.4 ± 1.2 vs. 2.3 ± 0.9; *Chi*^2^ = 7.04, *p* < 0.05) (Figure 7A). This significant decrease intensified during the sympto PET compared to CLD (0.4 ± 0.3 vs. 2.3 ± 0.9; *Chi*^2^ = 32.18, *p* < 0.001) and CHD PET scans (0.4 ± 0.3 vs. 1.4 ± 1.2; *Chi*^2^ = 9.11, *p* < 0.01) (Figure 7A). ^18^F-DOPA uptake in the Amyg significantly decreased during the sympto PET compared to CLD PET (1.9 ± 0.5 vs. 2.5 ± 0.7; *Chi*^2^ = 5.16, *p* < 0.05) (Figure 7A).

The comparison of ^18^F-DOPA uptake between the sympto PET and the CHD PET showed a significant decrease for all cerebral structures except the Amyg. However, in the basal ganglia, a greater decrease in ^18^F-DOPA uptake was observed in the Ant/Post CdN (around 95%: 94.2 ± 7.8) than in the Ant/Post putamen (around 75%: 74.1 ± 7.4), the V Str (around 50%: 48.3 ± 14.1) and the SN (around 25%: 24.0 ± 36.2). Similarly, in the cortical areas, a greater decrease of ^18^F-DOPA uptake was observed in the Ins (around 72%: 72.5 ± 24.7) than in the OFC (around 24%: 23.4 ± 28.6) and the ACC (around 63%: 63.1 ± 25.7).

#### Individual comparisons

**Figure 8:**
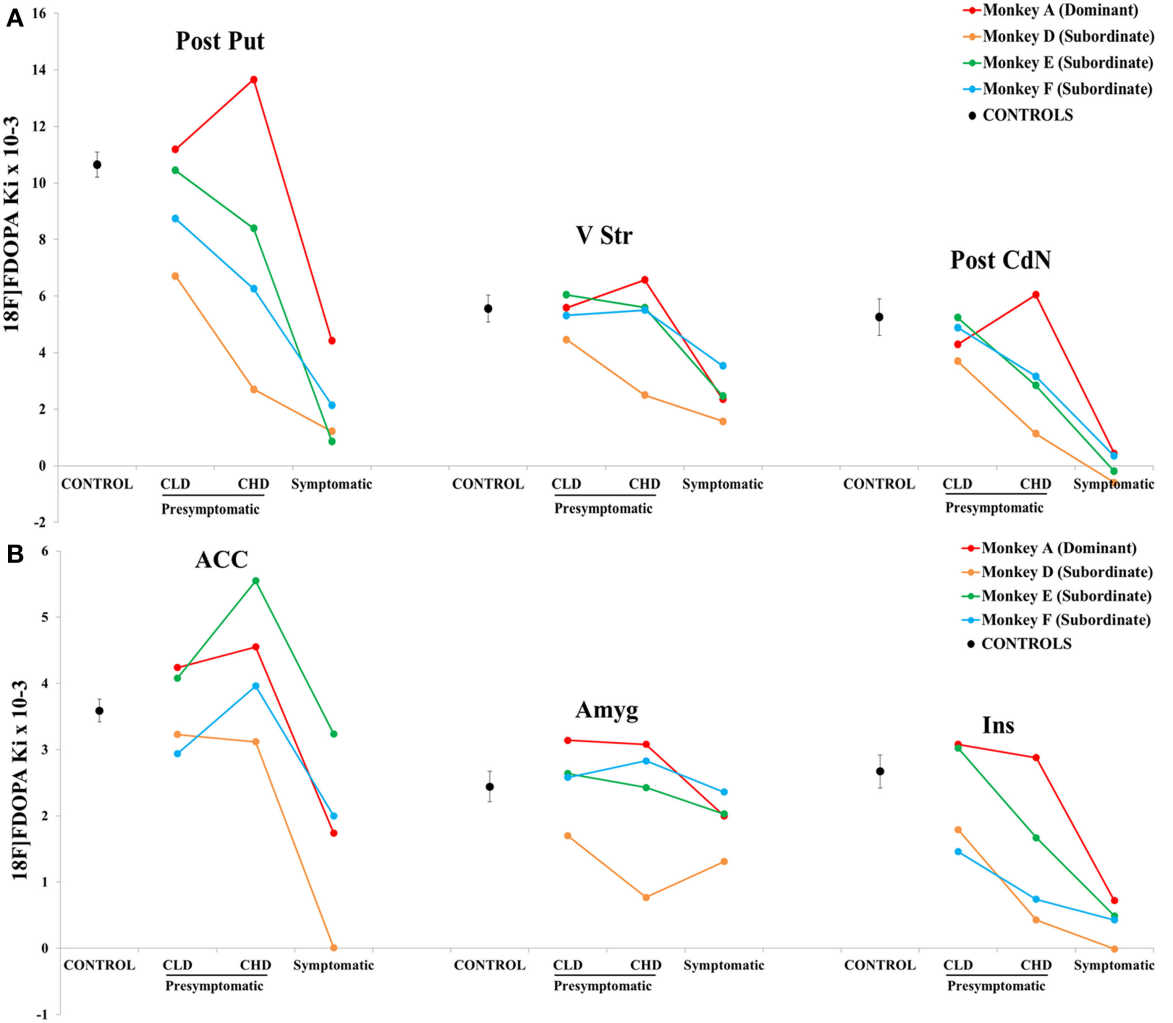
**Individual evolution of ^18^F-DOPA uptake in (A) 3 striatal (posterior caudate nucleus, posterior putamen and ventral striatum) and (B) 3 cortical (insula, amygdala and anterior cingulate cortex) areas (*n* = 4)**. The ^18^F-DOPA uptake value in the different motor states (presymptomatic and symptomatic) is represented for the most dominant (red line) and the three most subordinate animals (green, blue and orange lines). The ^18^F-DOPA uptake value for the control group is also shown (dotted line). The scale showed a difference and the lower threshold was 0.001.

Overall, the results showed a stronger inter-animal variability of the Ki uptake during the CLD and the CHD PET, with the subordinate monkey D showing the lowest Ki value in all structures (apart in the ACC and the Ins during the CLD PET) (Figures 8A,B). ^18^F-DOPA uptake decreased linearly in Post Put, Post CdN and Ins in all three subordinate animals, whereas the Ki uptake in the dominant animal only decreased during the symptomatic PET. A stronger individual variability of Ki was observed in ACC. However, the dynamic of change was comparable between the 4 animals apart for the subordinate monkey D, whose Ki uptake was not detectable during the sympto PET. Finally, ^18^F-DOPA uptake in V Str and Amyg did not change between CLD and CHD PETs, except for the subordinate monkey D. The only change involving a decrease in ^18^F-DOPA uptake in V Str and Amyg occurred during the sympto PET.

#### Correlation analyses

There was a negative correlation between the clinical score and the ^18^F-DOPA uptake in Ant CdN (*r* = −0.88, *p* < 0.001), Post CdN (*r* = −0.88, *p* < 001), Ant Put (*r* = −0.86, *p* < 0.001), Post Put (*r* = −0.79, *p* < 0.01), V Str (*r* = −0.75, *p* < 0.01), and Ins (*r* = −0.74, *p* < 0.01). Similarly, there was a negative correlation between the time spent in inactivity and the ^18^F-DOPA uptake in Ant CdN (*r* = −0.88, *p* < 0.05), Post CdN (*r* = −0.64, *p* < 0.05), Ant Put (*r* = −0.71, *p* < 0.05), Post Put (*r* = −0.62, *p* < 0.05).

The success rate on detour trials was positively correlated to the ^18^F-DOPA uptake in Ant CdN (*r* = 0.77, *p* < 0.05), Post CdN (*r* = 0.74, *p* < 0.05), Ant Put (*r* = 0.79, *p* < 0.05), Post Put (*r* = 0.76, *p* < 0.05), ACC (*r* = 0.77, *p* < 0.05) and Ins (*r* = 0.69, *p* < 0.05). Moreover, there was a negative correlation between the percentage of errors responses in the detour trials and the ^18^F-DOPA uptake in Ant CdN (*r* = 0.70, *p* < 0.05), Ant Put (*r* = 0.74, *p* < 0.01), Post Put (*r* = 0.74, *p* < 0.01), Ins (*r* = 0.69, *p* < 0.05) and ACC (*r* = 0.77, *p* < 0.01). Finally, a positive correlation was observed between the frequency of emitted affiliative behaviors and the ^18^F-DOPA uptake in Ins (*r* = 0.67, *p* < 0.05).

## Discussion

The aim of this study was to focus on social behavioral changes within a group of female long-tailed macaques after chronic administration of low (CLD) and then high (CHD) doses of MPTP. These MPTP administration protocols made it possible to evaluate each individual in three motor states (normal, presymptomatic and symptomatic).

Following the CLD-MPTP protocol, a significant increase was highlighted in the frequency of aggressive and affiliative behaviors from the baseline state to the CLD-presymptomatic motor state. Indeed, a higher number of conflicts followed by reconciliation events were observed. More precisely, these conflicts occurring during the CLD-MPTP protocol were very intense with slapping, biting and other aggressive behaviors involving direct contact between individuals. None of these aggressive behaviors were observed in the baseline state, where the few conflicts observed were of low intensity and mainly limited to visual threats with the extensive use of facial expressions. Interestingly, these social behavioral changes were only shown by the subordinate animals. Moreover, a temporary change of the dominance hierarchy was observed; thus, monkey D presented the lowest hierarchical status compared to monkeys E and F. Individual data within this subgroup were too heterogeneous to show any significant variations in the three categories of social behaviors. Indeed, only the frequency of emitted affiliative behaviors increased significantly in the CLD-presymptomatic motor state compared to the baseline state. A clearer picture could be obtained by increasing the number of animals in this study, taking the hierarchical status into consideration.

From the CLD-presymptomatic to the CHD-presymptomatic motor states, the frequency of social behaviors decreased. The dominance hierarchy was similar to the baseline state; thus monkey D recovered its initial hierarchical status. At this time, quantitative data analysis in PET scans made it possible to highlight a dopaminergic denervation in two brain structures, the insula and the posterior caudate nucleus, which could therefore be involved in the social behavioral changes observed from the CLD-presymptomatic motor state to the CHD-presymptomatic motor state.

Finally, during the symptomatic motor state, the frequency of social behaviors was lower across all categories, than the frequency observed in the CLD and the CHD-presymptomatic motor states. The dominance hierarchy was not modified and was similar to that observed during the baseline state. Motor and cognitive disorders were also observed in this stable-symptomatic state. PET scans results then showed a dopaminergic denervation in all the evaluated cortical and subcortical structures.

In the present study, the choice was made to intoxicate not just one animal with MPTP, but all the animals in the group. This decision was made in order to increase the probability of observing social behavioral changes due to inter-individual variability of sensitivity to MPTP in monkeys. Moreover, this study did not use a control group to compare the distribution of interactions over time in the present study group. This is explained by the fact that the stability of primate groups can vary despite similar sizes and similar ecological conditions (Sueur et al., 2011) and the distribution of grooming can differ considerably between groups (Perry, 1996; Manson et al., 1999; Dufour et al., 2011). Consequently, it is more efficient to analyze social relationships of a single group over time when investigating network stability or instability. For example, this enables us to compare different observation periods where breeding conditions have changed, and study how individuals within one same group cope with perturbation (Dufour et al., 2011). The present study examines the evolution of social interactions over time in order to understand how our group of female macaques coped socially with disease-related changes.

### Chronic low- and high-dose protocol is effective to observe social behavioral changes and dissociate them from cognitive and motor impairments

This is the first study describing social behavioral changes during the presymptomatic motor state following MPTP administration before the onset of cognitive and motor disorders. On the one hand, a previous study has also shown social behaviors changes (aggressive and affiliative) during the symptomatic motor state induced by MPTP administration (hemiparkinsonian model in vervet monkey model) (Melega et al., 1996). However, this study was performed during the symptomatic motor state. Thus, it is difficult to dissociate the effect of motor disorders on social behavioral changes. On the other hand, it is important to note that two previous studies in the CLD-MPTP-treated macaque model showed cognitive impairment in the presymptomatic motor state (Schneider and Pope-Coleman, 1995; Vezoli et al., 2011), which was not the case in the present study. Indeed, cognitive and motor disturbances appeared only during the symptomatic motor state following the CHD-MPTP protocol, and their occurrence was almost simultaneous. Thus, it was difficult to really dissociate the impact of motor disorders on cognitive abilities. Several hypotheses could explain the differences in cognition results between this study and the two studies mentioned above. Firstly, the dose of MPTP administered to animals was different: 0.1 mg/kg in the present study vs. 0.05–0.075 mg/kg in Schneider's study and 0.2 mg/kg in Vezoli's study. Secondly, MPTP was administered every 4–5 days for more than 50 weeks in the present study vs. 2–3 times per week for 24 weeks in Schneider's study and every 3–4 days for 5–25 weeks in Vezoli's study. Thirdly, the social housing could also have an impact (Prescott et al., 2010), as a possible protective effect against development of cognitive deficits. Finally, the sex and age of animals differ between studies (Prescott et al., 2010; Darusman et al., 2014). Moreover, over-training might have an impact on such simple task as it was shown to be associated with less engagement of cognitive control areas, which is specifically tested with this task (Patel et al., 2013). In the present study, there was an apparent gain in performance in CLD-presymptomatic motor state compared to baseline state; this might indicate that animals maintained learning capacities on the task with the MPTP regimen used and the task chosen in the present study.

### Heterogeneity of social behavioral changes according to hierarchical status

In the present study, social behavioral changes were only observed in the subordinate subgroup when compared to the dominant subgroup. It was also important to note that the most dominant individual was also involved in post-conflicts reconciliation events, suggesting that it retained its regulatory role within the social group (Petit and Thierry, 1992).

Hierarchical status was assessed using methods that have been validated and widely used in ethology (David, 1987, 1988; Kaplan et al., 2002). Several studies have already demonstrated the specific role played by dopamine in hierarchical status even if other neurotransmitters such as noradrenalin and serotonin have been implicated in behavioral disorders (Raleigh et al., 1991; Siever, 2008; Krämer et al., 2011). A study by Kaplan et al. (Kaplan et al., 2002) has shown that in non-human primates, a higher concentration of homovanillic acid (dopamine's catabolite) was found in the cerebrospinal fluid of dominant animals than in subordinate animals, whatever their gender. PET imaging studies with a specific D_2_/D_3_ receptor radiotracer also highlighted differences in radiotracer uptake according to hierarchical status in male long-tailed monkeys (Morgan et al., 2002; Nader et al., 2012) and in humans (Martinez et al., 2010). Interestingly, levels of D_2_/D_3_ receptor availability appeared to be sensitive to changes in housing conditions. This sensitivity was such that the transition from individual to social housing resulted in significant increases in D_2_/D_3_ levels in dominant male animals, whilst subordinate animals showed no change (Morgan et al., 2002). Nader et al. recently extended their earlier work in male long-tailed monkeys to female long-tailed monkeys, with the additional use of a dopamine transporter (DAT) radiotracer (Nader et al., 2012). Their results showed that although neither DAT nor D_2_/D_3_ receptor availability in the caudate nucleus and putamen was predictive of social rank, both significantly changed after the formation of social hierarchies in female long-tailed monkeys. Dopamine D_2_/D_3_ receptor availability significantly increased in females that became dominant, whereas DAT availability decreased in subordinate females. Otherwise, an association between the dopamine D_4_ receptor and a personality trait such as novelty seeking has been demonstrated in both humans (Benjamin et al., 1996; Ebstein et al., 1996) and non-human primates (Bailey et al., 2007). In the present study, the role of dopamine in the behavioral changes observed can be evaluated through the use of ^18^F-Dopa radiotracer, a specific radiotracer of dopaminergic innervation, during PET scans.

### A possible association between dopaminergic denervation and social behavioral changes

During the CLD-presymptomatic motor state following the CLD-MPTP protocol, whereas a significant increase of the frequency of social behaviors compared to the baseline state was observed, no significant difference was identified between the Ki values of ^18^F-Dopa uptake in the MPTP-treated animals compared to that of the control animals. However, as MPTP-treated animals were not their own control, it was impossible to know if they really present any variation of their Ki values of ^18^F-Dopa uptake. Nevertheless, it was interesting to note that two subordinate animals (monkeys D and F) had lower Ki uptake values in the insula and dorsal putamen than those observed in control individuals. Recent studies have shown that neural circuits (Noonan et al., 2014) and dopaminergic innervation (Nader et al., 2012) could differ from dominant to subordinate individuals. Thus, a brain structure with initially low Ki values would probably be more sensitive to MPTP intoxication and would therefore show dopaminergic denervation earlier than other brain structures. However, MPTP sensitivity was not seen to differ according to hierarchical status in this study. Finally, the use of non-dopaminergic radiotracer, including namely serotoninergic radiotracers, would have probably shown interesting and complementary results to the present study (Raleigh et al., 1991; Siever, 2008; Krämer et al., 2011). Indeed, a study in male vervet monkeys has shown that there was a distinction between dominance and aggression and has strongly suggested that when hierarchical relationships are uncertain, serotonergic mechanisms may mediate the behaviors which permit a male to attain high dominance status (Raleigh et al., 1991). Moreover, whereas previous reports have suggested an inverse relationship between serotonin level and aggressive behavior with low levels of serotonin leading to higher aggression and vice versa, such a simple relationship seemed to be inconsistent with the current data obtained in the study Krämer et al. (2011).

During the CHD-presymptomatic motor state following the CHD-MPTP protocol, a significant decrease of the frequency of social behaviors compared to the CLD-presymptomatic motor state was observed. Moreover, a dopaminergic denervation in the insula and the posterior caudate was identified, only in the three subordinate animals but not in the dominant animal.

The insula and the caudate nucleus are both involved in the organization of the social behavioral network or “social brain network,” which also includes the prefrontal cortex, the amygdala, the hippocampus, the medial preoptic area, the hypothalamus, the anterior cingulate cortex and the basal ganglia as a whole (Newman, 1999; Skuse and Gallagher, 2009). This network is also closely linked to the reward circuit (O'Connell and Hofmann, 2011). The two abovementioned networks are involved in the regulation of emotions and aggression (Davidson et al., 2000; Siever, 2008).

More specifically, the insula plays a role in the regulation and expression of several emotions, including disgust (Lamm and Singer, 2010; Jezzini et al., 2012), an emotion that is particularly impaired in parkinsonian patients (Suzuki et al., 2006), as well as the expression of social behavior. A recent study in rhesus macaques used intracortical microstimulations in different regions of the insula highlight its involvement in the expression of disgust and in the expression of an essential affiliative facial expression in social relationships in macaques, the “lipsmacking display” (Caruana et al., 2011). Thus, an impairment of the insula during the CHD-presymptomatic motor state could be associated with the impaired expression of affiliative behaviors, as suggested by the positive correlation observed in our study between the frequency of emitted affiliative behaviors and the ^18^F-DOPA uptake in the insula.

The caudate nucleus is particularly involved in the human “trust network” (Baumgartner et al., 2008), and its activity increases throughout cooperation between protagonists (De Dreu, 2012). An impairment of the caudate nucleus during the presymptomatic motor state could therefore be associated with an impairment of social relationships. This would explain why the frequency of social behaviors decreased from the CLD to the CHD presymptomatic motor states. This decrease could be associated with a “loss of emotional interest,” and could also correspond to an “apathetic state” during the presymptomatic motor state. Indeed, apathy is characterized in humans by a loss of interest and a lower participation in usual activities, a lack of initiative, diminished initiated activities, an emotional indifference and a flat affect. Moreover, apathy can be described in 3 dimensions (Levy and Dubois, 2006): cognitive, emotional and behavioral. However, the lack of effect on the ORDT performance during the CHD-presymptomatic motor state excluded the cognitive dimension (Brown et al., 2012) and argued to possible emotional and/or behavioral attempt.

Finally, and surprisingly, other cortical structures belonging to the social behavior network, including the orbitofrontal cortex, the anterior cingulate cortex, amygdala, and a limbic structure of the basal ganglia, the ventral striatum, did not seem to be involved in behavioral changes observed during the presymptomatic motor states. Indeed, these structures were only impaired during the stable-symptomatic motor state and could be associated not only with social behavioral changes but also with cognitive and motor disorders, as suggested by the correlations observed between the Ki value of several cerebral structures and motor/cognitive disorders. This suggests that the dopaminergic innervations of these brain structures are less sensitive to MPTP in our experimental conditions.

## Conclusion

In conclusion, the present study of the MPTP-treated non-human primate model of Parkinson's disease showed that behavioral changes appeared early, before the onset of motor and cognitive disorders. This could be compared with clinical data obtained in parkinsonian patients showing a high incidence of behavioral changes in the early stages of the disease (Aarsland et al., 2009). Furthermore, these behavioral changes were most common among subordinate individuals. Finally, the results of the present study revealed the early sensitivity of cortical structures such as the insula to MPTP. However, additional studies including more animals to limit the inter-individual variability would be necessary to clarify the role of hierarchical status on behavioral changes. Likewise, the use of non-dopaminergic radiotracer, including namely serotoninergic radiotracers, would have probably shown interesting and complementary results to the present study.

### Conflict of interest statement

The authors declare that the research was conducted in the absence of any commercial or financial relationships that could be construed as a potential conflict of interest.
