# Supplementary material for: Social behavioral changes in MPTP-treated monkey model of Parkinson's disease
Source: Front Behav Neurosci. 2015 Feb 26;9:42. doi: 10.3389/fnbeh.2015.00042 (PMC4341564; doi:10.3389/fnbeh.2015.00042)

**SUPPLEMENTARY DATA**

**ORDT EXPERIMENTAL SETUP**

Diagrammatic representation shows the position of the box on the tray, the reward in the box, and the open side of the box on the 35 trials making up each test session. In 20 of these configurations, a closed (transparent) side of the box was facing the subject, making a detour (to the left for 1-10 and to the right for 11-20) necessary to retrieve the reward (called "difficult" or "detour" trials). In 15 of these configurations (5, 6, 7, 12, 13, 14, 19, 20, 21, 26, 27, 28, 33, 34, 35), the open side of the box was facing the subject so that no detour was required to reach the reward (called the "easy" trial).


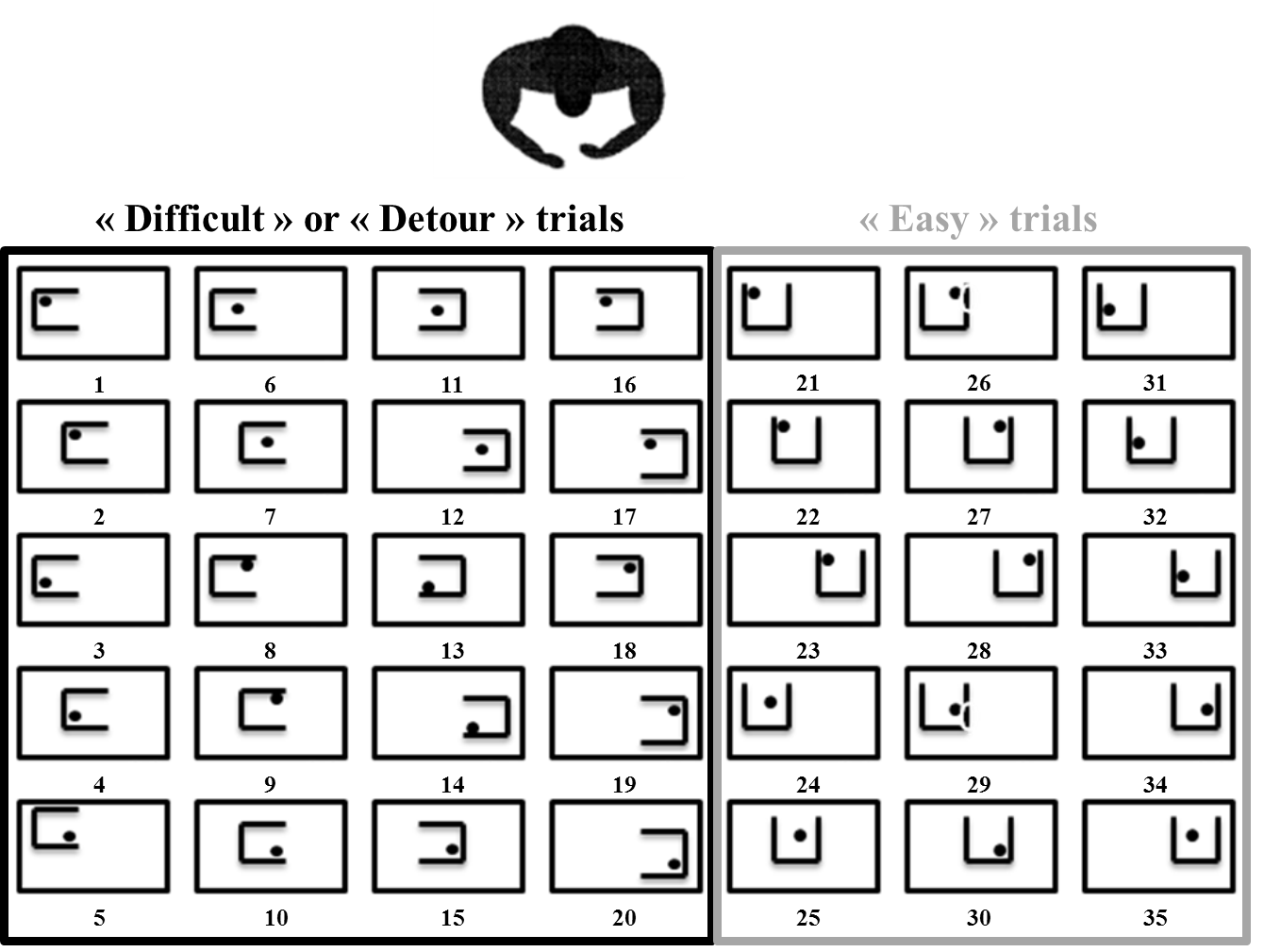

Supplement: Supplementary file 1 [file Table1.DOCX]
